# Supplementary material for: Rapidly making biodegradable and recyclable paper plastic based on microwave radiation driven dynamic carbamate chemistry
Source: Nat Commun. 2025 Jul 15;16:6523. doi: 10.1038/s41467-025-61722-0 (PMC12264110; doi:10.1038/s41467-025-61722-0)
Supplement: Supplementary file 1 — Supplementary Information [file 41467_2025_61722_MOESM1_ESM.pdf]

Supplementary Information for

Rapidly Making Biodegradable and Recyclable Paper Plastic Based on Microwave Radiation Driven Dynamic Carbamate Chemistry

Xinxin Yang[1, 2, 3#], Le Yu[2#], Bowen Zhang[3], Yongheng Wang[4], Xiangzheng Jia[4], Erlantz Lizundia[5], Chang Chen[6], Fuhao Dong[3], Luhe Qi[2], Lu Chen[2], Enlai Gao[4], Xu Xu[1]*, He Liu[3]* & Chaoji Chen[2]**

[1] *National Key Laboratory for Development and Utilization of Forest Food Resources, Jiangsu Co-Innovation Center of Efficient Processing and Utilization of Forest Resources, College of Chemical Engineering, Nanjing Forestry University, Nanjing 210037, China.*

[2] *Hubei Biomass-Resource Chemistry and Environmental Biotechnology Key Laboratory, Hubei Provincial Engineering Research Center of Emerging Functional Coating Materials, School of Resource and Environmental Sciences, Wuhan University, Wuhan 430079, China.*

[3] *National Key Laboratory for Development and Utilization of Forest Food Resources, Institute of Chemical Industry of Forest Products, Chinese Academy of Forestry, Nanjing 210042, China.*

[4] *Department of Engineering Mechanics, School of Civil Engineering, State Key Laboratory of Water Resources and Hydropower Engineering Science, Wuhan University, Wuhan 430072, China*

[5] *Life Cycle Thinking Group, Department of Graphic Design and Engineering Projects, University of the Basque Country (UPV/EHU), 48013 Bilbao, Spain*

[6] *State Environmental Protection Key Laboratory of Soil Health and Green Remediation, Huazhong Agricultural University, Wuhan 430070, China*

[#] *Equal contributions.*

* *Corresponding authors: Xu Xu, email: xuxu200121@njfu.edu.cn; He Liu, email: liuhe.caf@gmail.com; Enlai Gao, email: enlaigao@whu.edu.cn; Chaoji Chen, email: chenchaojili@whu.edu.cn.*

This file includes

Supplementary Notes

Suppl. Table. 1. Formulations of the raw materials.

Suppl. Table. 2. Coarse-grained parameters of cellulose and polymer microfibrils.

Suppl. Table. 3. Classification System for Skin Reaction.

Suppl. Table. 4. Primary irritation index categories in a rabbit.

Suppl. Table. 5. Dermal observations of skin irritation test.

Suppl. Table. 6. Magnusson and Kligman scale.

Suppl. Table. 7. Guinea pig sensitization dermal reactions.

Suppl. Table. 8. Guinea pig sensitization dermal reactions (results of positive control test).

Suppl. Table. 9. Relevant detailed information about the experimental animals.

Suppl. Table. 10. Input and output of the in situ lignin regeneration treatment method presented in this work.

Suppl. Table. 11. Density and tensile strength used to normalize the environmental impacts.

Suppl. Table. 12. Summary of microplastic characteristic fragments, quantitative ions, standard curves, R^2 and limits of quantification produced by 11 standard plastic products.

Suppl. Table. 13. Summary of microplastics test results for paper plastics.

Suppl. Fig. 1. Schematic diagram of the dynamic carbamate chemistry.

Suppl. Fig. 2. Synthesis of cyclic-carbonated soybean oil (CSBO).

Suppl. Fig. 3. Synthesis of acrylpimaric acid cyclic carbonate (APAC).

Suppl. Fig. 4. FTIR spectra of raw materials.

Suppl. Fig. 5. ^{1}H NMR spectra of the raw material (cyclic-carbonated soybean oil (CSBO)).

Suppl. Fig. 6. NMR spectra of the raw materials (cyclic carbonate APAC).

Suppl. Fig. 7. Radar plots comparing the performance of commercial plastics, in which the results are normalized by the maximum value of each characteristic.

Suppl. Fig. 8. Image analysis to reveal the morphologies of the fracture cross section in paper and paper plastic.

Suppl. Fig. 9. XRD patterns of paper and paper plastic.

Suppl. Fig. 10. ^{13}C NMR spectra of paper and paper plastic.

Suppl. Fig. 11. Structures in all-atom molecular dynamics simulations.

Suppl. Fig. 12. Tensile stress–strain curve of NIPU (CSBO/APAC-PEI).

Suppl. Fig. 13. Paper plastic strip (three layers) with a width of 100 mm and thickness of 0.12 mm

can withstand a 65 kg boy.

Suppl. Fig. 14. SAXS tests of paper and paper plastic.

Suppl. Fig. 15. Micro-morphology images of silicone rubber.

Suppl. Fig. 16. Tensile stress–strain curve of silicone rubber modified paper (SR/R composite). The inset corresponds to the tensile stress–strain curve of silicone rubber.

Suppl. Fig. 17. Density changes and Specific tensile strength of different paper types before and after preparation into paper plastics.

Suppl. Fig. 18. Moving patterns of microfibrils under uniaxial stretching.

Suppl. Fig. 19. Load-transfer during stretching.

Suppl. Fig. 20. Images of the fracture appearance of the paper and paper plastic after tensile tests.

Suppl. Fig. 21. The photographs of different types of paper prepared into paper plastic.

Suppl. Fig. 22. Mechanical properties of paper plastics before and after modification.

Suppl. Fig. 23. Thermal stability of epoxy and polyurethanes (PU).

Suppl. Fig. 24. Thermal stability.

Suppl. Fig. 25. 2D and 3D TG–FTIR spectra, as a function of FTIR wavenumber and TG temperature.

Suppl. Fig. 26. Analysis of water and solvent resistance.

Suppl. Fig. 27. The morphology of paper plastic board.

Suppl. Fig. 28. Comparative images of heavy loads. a, paper plastic. b, NIPU.

Suppl. Fig. 29. Schematic illustration of the bag preparation process using the paper plastic.

Suppl. Fig. 30. The schematic diagram of the welding process of two plastic strips and the images after stretching of the welding sample.

Suppl. Fig. 31. The products prepared by paper plastic and the solution resistance of the products.

Suppl. Fig. 32. Paper plastics stained with dye in different colors.

Suppl. Fig. 33. The barrier properties of paper plastic and commercial plastics.

Suppl. Fig. 34. In vitro cytotoxicity test of paper plastic.

Suppl. Fig. 35. Degradation process of paper and NIPU buried in soil (Nanjing).

Suppl. Fig. 36. Determination of the ultimate aerobic biodegradability of paper plastics under controlled composting conditions.

Suppl. Fig. 37. Exploring whether paper plastics produce microplastics.

Suppl. Fig. 38. Recyclability Test of Pure Polymer NIPU.

Suppl. Fig. 39. Comparison of the climate change potential of paper plastic to a variety of benchmark plastic films and composite materials according to life cycle assessment (LCA).

Suppl. Fig. 40. Environmental Impact Contribution of Each Unit in Paper Plastic Production.

Supplementary References S1–S9

Supplementary Note 1: Preparation of APAC/CSBO–PEI non-isocyanate polyurethane (NIPU).

The raw material cyclic-carbonated soybean oil (CSBO) was prepared according to our previous work. CSBO, APAC, and PEI were stirred at room temperature to obtain a homogeneous mixture, which was poured into a Teflon mold and cured at 80 °C for 6 h to receive APAC/CSBO–PEI non-isocyanate polyurethane (NIPU).

Supplementary Note 2: Theoretical calculation of biobased carbon content in paper plastics.

CSBO ($C_{61}H_{102}O_{18}$) is prepared by the reaction of ESO ($C_{57}H_{102}O_{10}$, containing 4 epoxy groups in the structure) and CO_2, so the bio-based carbon content of CSBO without CO_2 is 93.44% ($57/61 \times 100\%$). In addition, APADE ($C_{23}H_{34}O_4$, containing 3 non-bio-based carbons derived from acrylic acid) was reacted with epichlorohydrin to obtain APADE ($C_{29}H_{42}O_6$, and 6 non-bio-based carbons derived from epichlorohydrin were introduced), and then APADE was reacted with CO_2 to obtain APAC ($C_{31}H_{42}O_{10}$). Therefore, the carbon content of CO_2 introduced into APAC is 6.45% ($2/31 \times 100\%$) , and the bio-based carbon content excluding CO_2 is 64.52% ($(31 - 2 - 6 - 3)/31 \times 100\%$).

In the preparation process of paper plastics, the mass ratio of each component is 31: 5: 14: 50 (CABO: APAC: PEI: paper). Therefore, the theoretical bio-based carbon content of paper plastic without CO_2 is: $0.31 \times 93.44\% + 0.05 \times 64.52\% + 0.5 \times 100\% = 82.2\%$; the theoretical bio-based carbon content of paper plastic with CO_2 is: $0.31 \times 100\% + 0.05 \times (64.52\% + 6.45\%) + 0.5 \times 100\% = 84.6\%$.

The theoretical renewable CO_2 content (considering biogenic carbon and CO_2 that can be potentially captured from the atmosphere) of paper plastic is 1.754 kg. This value is obtained as:

- CSBO: biogenic carbon content in 1 kg of soybean oil is 0.79 kg + biogenic carbon content in 1 kg of ascorbic acid is 0.41 kg + 54.06 kg CO_2 per 318 kg of final material. 3.005 kg CO_2 per kg of CSBO.

- APAC: biogenic carbon content in 1 kg of ascorbic acid is 0.41 kg + 8 kg CO_2 per 52 kg of

final material. 0.164 kg CO_2 per kg of APAC.

- 0% in polyethyleneimine.
- 44% in paper from the cellulosic molecule. 1.628 kg CO_2 per kg of paper.

Supplementary Note 3: Environmental impacts of the paper plastic compared to benchmark plastic films and composite materials.

The Life Cycle Inventory (LCI) data of the upstream production of chemicals, electricity, and water were collected from the ecoinvent v3.11 database and literature. The LCI data for diglycidyl ester of acrylpimaric acid production was estimated based on the process that uses acrylic acid and epichlorohydrin as feedstock. The electricity consumption for the 4 h reaction at 117 ºC is estimated to be 1.72 kWh using an Asynt ADS-HP-NT stir plate with a device power of 616 W (https://www.asynt.com/wp-content/uploads/2011/07/Asynt-Hotplate-Manual-V1.0.pdf) at a 70% workload. Filtering (0.1 h) and vacuum drying is needed (1 h vacuum drying) for the final material. The electricity consumption of 4.2 Wh is modeled for filtering using a Buchner filtration with a diaphragm pump having a power of 60 W (https://assets.fishersci.com/TFS-Assets/CCG/EU/Welch-Vacuum-Technology/brochures/12139%20FB%20Vacuum%20pumps_EN.pdf) at a 70% workload. For the drying, the electricity consumption of 1.15 kWh is modeled using an oven with a power of 1400 W equipped with a 248 W pump at a 70% workload.

The LCI for tetrabutylammonium iodide (TBAI) production was estimated based on a commercial process that reacts tributylamine with 1-iodobutane. Briefly, the reaction follows:

$$NBu^3 + BuI \rightarrow [NBu_4^+][I^-]$$

The reaction takes place in acetonitrile (1.5 L) at 50 ºC for 18 h. The electricity consumption of 7.76 kWh is modeled using an Asynt ADS-HP-NT stir plate with a device power of 616 W (https://www.asynt.com/wp-content/uploads/2011/07/Asynt-Hotplate-Manual-V1.0.pdf) at a 70% workload. Hexane (0.5 L), and ethanol (1 L) are added for washing and recrystallization. Filtering (0.1 h) and vacuum drying is needed (12 h vacuum drying) for the final material. The electricity

consumption of 4.2 Wh is modeled for filtering using a Buchner filtration with a diaphragm pump having a power of 60 W (https://assets.fishersci.com/TFS-Assets/CCG/EU/Welch-Vacuum-Technology/brochures/12139%20FB%20Vacuum%20pumps_EN.pdf) at a 70% workload. For the drying, the electricity consumption of 13.84 kWh is modeled using an oven with a power of 1400 W equipped with a 248 W pump at a 70% workload. Methyl iodide (CH_3I) is utilized as a proxy material for 1-iodobutane due to lack of information in the database utilized (they are structurally similar and a similar impact is expected), while iodine (I_2) is used to balance the iodine content. In summary, 1 kg of TBAI is modeled with 630 g of CH_3I and 420 g of I_2 (5 wt% losses considered during the synthesis). Generated waste is treated as hazardous solvent.

The LCI data for polyethyleneimine production was estimated based on the process that uses monoethanolamine, hydrogen chloride, chlorosulfuric acid and sodium hydroxide as feedstock. Ethyleneimine is first formed, followed by its polymerization. Monoethanolamine is reacted with HCl and the resulting product is treated with chlorosulfuric acid (sulfuric acid is used as a proxy material due to lack of data in the database). The polymerization process with NaOH as a base catalyst by ring opening polymerization. A 95 % reaction yield is estimated.

The LCIAs of the paper plastic was first conducted for 1000 kg of paper plastic, and then the results were converted based on a second FU that takes the tensile strength into consideration, as shown in Eq. (1).

$$EI'_P = EI_p \times \frac{\rho_P}{\sigma_P} \qquad (1)$$

where EI'_P is the environmental impacts of the paper plastic using the second FU that was converted based on the density (ρ_P) and the tensile strength of the material (σ_P). EI_p is the environmental impact per 1000kg of paper plastic. The ratio of density to tensile strength is used in the second FU as it considers the impacts of material properties on the functionality of the materials[1]. The density and tensile strength of the paper plastic in this study was measured to be 1.245 g·cm^{-3} and 126 MPa, respectively. Obtained environmental impacts were compared to benchmark plastic films and composite materials according to the ecoinvent v3.11 database and the Environmental Footprint 3.1 assessment method. When the data corresponding to the film was

not available on the database, we implemented a combination of plastic production (granulate in most of the cases) with extrusion for a plastic film. The entries selected are:

- packaging film production, low density polyethylene | packaging film, low density polyethylene | Cutoff, U - RoW
- polyurethane production, rigid foam | polyurethane, rigid foam | Cutoff, U - RER
- polyethylene terephthalate production, granulate, bottle grade | polyethylene terephthalate, granulate, bottle grade | Cutoff, U - RoW
- polylactic acid production, granulate | polylactic acid, granulate | Cutoff, U - GLO
- polypropylene production, granulate | polypropylene, granulate | Cutoff, U - RoW
- acrylonitrile-butadiene-styrene copolymer production | acrylonitrile-butadiene-styrene copolymer | Cutoff, U - RoW
- polybutadiene production | polybutadiene | Cutoff, U - RoW
- polycarbonate production | polycarbonate | Cutoff, U - RoW
- polyvinyl chloride production, unspecified polymerisation, weighted average | polyvinyl chloride, unspecified polymerisation, weighted average | Cutoff, U - RoW
- polyvinylfluoride production | polyvinylfluoride | Cutoff, U - RoW
- polyvinylidenchloride production, granulate | polyvinylidenchloride, granulate | Cutoff, U – RoW
- market for polymethyl methacrylate | polymethyl methacrylate | Cutoff, U - RoW
- glass fibre reinforced plastic production, polyamide, injection moulded | glass fibre reinforced plastic, polyamide, injection moulded | Cutoff, U - RoW
- carbon fibre reinforced plastic, injection moulded | carbon fibre reinforced plastic, injection moulded | Cutoff, U – GLO
- extrusion, plastic film | extrusion, plastic film | Cutoff, U - RoW

The end-of-life modeling includes the fossil-CO_2 emissions during combustion or biodegradation, together with the following processed have been selected:

- paper plastic: treatment of biowaste, industrial composting | compost | Cutoff, U - RoW
- benchmark plastic films and composite materials: treatment of waste plastic, mixture, municipal incineration | waste plastic, mixture | Cutoff, U - RoW

Furthermore, the density and tensile strength values in Supplementary Tab. 11 have been utilized to normalize the environmental impacts of benchmark plastic films and composite materials.

Supplementary Tab. 1 | Formulations of the raw materials.

Raw materials	Molar mass (M_w, g/mol)	Content (g)	Cyclic carbonate groups content (mol)	Amino groups content (mol)
APAC	574	50	0.174	–
CSBO	1122	310	1.105	–
PEI	1800	140	–	1.29
The molar ratio of cyclic carbonate group and amino group is about 1:1				

Supplementary Tab. 2 | Coarse-grained parameters of cellulose and polymer microfibrils.

Microfibrils	d_0 (nm)	Y (GPa)	k_d (k_BT/Å^2)	Persistence length (μm)	Interchain interaction energy per bead (k_BT)	
cellulose	3	20	115	1	cellulose/cellulose	84
					cellulose/NIPU	2×10^3
NIPU	3	15	86	1.5	NIPU/NIPU	40

Supplementary Tab. 3 | Classification System for Skin Reaction.

Erythema and Eschar Formation	Irritation score	Oedema Formation	Irritation score
No erythema	0	No oedema	0
Very slight erythema (barely perceptible)	1	Very slight oedema (barely perceptible)	1
Well-defined erythema	2	Well-defined oedema (edges of area well-defined by definite raising)	2
Moderate erythema	3	Moderate oedema (raised approximately 1 mm)	3
Severe erythema (beet redness) to eschar formation preventing grading of erythema	4	Severe edema (raised more than 1 mm and extending beyond exposure area)	4
Maximal possible score for irritation			8

Supplementary Tab. 4 | Primary irritation index categories in a rabbit.

Mean score	Response category
0–0.4	Negligible
0.5–1.9	Slight
2.0–4.9	Moderate
5–8	Severe

Supplementary Tab. 5 | Dermal observations of skin irritation test.

Extraction solvent	Rabbit No.	Group		Interval		
				**24 h**	**48 h**	**72 h**
0.9% NaCl injection (Polar test)	J1501	Test article	Erythema	0	0	0
			Oedema	0	0	0
		Negative control	Erythema	0	0	0
			Oedema	0	0	0
	J1502	Test article	Erythema	0	0	0
			Oedema	0	0	0
		Negative control	Erythema	0	0	0
			Oedema	0	0	0
	J1503	Test article	Erythema	0	0	0
			Oedema	0	0	0
		Negative control	Erythema	0	0	0
			Oedema	0	0	0
Sesame oil (Non-polar test)	F1501	Test article	Erythema	0	0	0
			Oedema	0	0	0
		Negative control	Erythema	0	0	0
			Oedema	0	0	0
	F1502	Test article	Erythema	0	0	0
			Oedema	0	0	0
		Negative control	Erythema	0	0	0
			Oedema	0	0	0
	F1503	Test article	Erythema	0	0	0
			Oedema	0	0	0
		Negative control	Erythema	0	0	0
			Oedema	0	0	0

Supplementary Tab. 6 | Magnusson and Kligman scale.

Patch test reaction	Grading scale
No visible change	0
Discrete or patchy erythema	1
Moderate and confluent erythema	2
Intense erythema and/or swelling	3

Supplementary Tab. 7 | Guinea pig sensitization dermal reactions.

Extraction solvent	Group	Animal Number	Excitation patch removed		Positive rate after challenge phase	Weight Range before injection (g)	Weight range after test (g)	Abnormal appearance except dermal reactions
			24 h	48 h				
0.9% NaCl injection (Polar test)	Control	J1001	0	0	0%	339.8–357.2	482.5–523.9	None
		J1002	0	0				None
		J1003	0	0				None
		J1004	0	0				None
		J1005	0	0				None
	Test	J2001	0	0	0%	335.3–356.9	470.9–522.6	None
		J2002	0	0				None
		J2003	0	0				None
		J2004	0	0				None
		J2005	0	0				None
		J2006	0	0				None
		J2007	0	0				None
		J2008	0	0				None
		J2009	0	0				None
		J2010	0	0				None
Sesame oil (Non-polar test)	Control	F1001	0	0	0%	337.1–362.7	492.6–521.9	None
		F1002	0	0				None
		F1003	0	0				None
		F1004	0	0				None
		F1005	0	0				None
	Test	F2001	0	0	0%	341.1–365.0	475.9–515.7	None
		F2002	0	0				None
		F2003	0	0				None
		F2004	0	0				None
		F2005	0	0				None
		F2006	0	0				None

		F2007	0	0				None
		F2008	0	0				None
		F2009	0	0				None
		F2010	0	0				None

Supplementary Tab. 8 | Guinea pig sensitization dermal reactions (results of positive control test).

Group	Animal Number	Excitation patch removed		Positive rate after challenge phase	Weight Range before injection (g)	Weight range after test (g)	Abnormal appearance except dermal reactions
		24 h	48 h				
Control	X1001	0	0	0%	323.7–355.0	476.4–520.7	None
	X1002	0	0				None
	X1003	0	0				None
	X1004	0	0				None
	X1005	0	0				None
Test	X2001	3	3	100%	330.4–364.7	456.9–472.3	None
	X2002	3	3				None
	X2003	3	3				None
	X2004	3	2				None
	X2005	3	2				None

Supplementary Tab. 9 | Relevant detailed information about the experimental animals.

	Skin irritation	Skin sensitization
Species	New Zealand white rabbit	White guinea pig
Number	6 (3 for polar test group and 3for non-polar group)	30, 15 for polar extract group and 15 for non-polar extract group (10 for test and 5 for control in each group)
Sex	Female	Male
Weight	Initial body weight not less than 2.0 kg	N/A
Health status	Healthy, young adult, nulliparous and not pregnant	Healthy, not previously used in other experimental procedures
Housing	Animals were housed in groups in cages (stainless steel cage, Suzhou Fengqiao purification equipment Co., Ltd.) identified by a card indicating the lab number, test code and first treatment date	Animals were housed in groups in cages (plastic cage, Suzhou Fengqiao purification equipment Co., Ltd.) identified by a card indicating the lab number and test code
Quarantine period	3 days, only healthy animals were selected	3 days, only healthy animals were selected
Animal purchase	Provided by Danyang Changyi experimental animal breeding Co., Ltd (Permit Code: SCXK (SU) 2021-0002)	Provided by Suzhou Hi-tech Zone Zhenhu Laboratory Animal Technology Co., Ltd (Permit Code: SCXK (SU) 2020-0007)
Feed	Rabbit Diet, Beijing Keao Xieli Feed Co., Ltd.	Guinea Pig Diet, Beijing Keao Xieli Feed Co., Ltd.
Water	Drinking water met the Standards for Drinking Water Quality (GB 5749-2006)	Drinking water met the Standards for Drinking Water Quality (GB 5749-2006)
Environment	Temperature 16-26 °C, Relative	Temperature 18-29 °C, Relative

	humidity 40%-70%, Lights 12 hours light/dark cycle	humidity 40%-70%, Lights 12 hours light/dark cycle
Fate of the animals after the in vivo experiment	Continue use after taking a break for at least 1 week	Euthanasia by CO_2 inhalation
Personnel	Associates involved were appropriately qualified and trained	Associates involved were appropriately qualified and trained
Veterinarian	Vet takes care of the whole course	Vet takes care of the whole course
Ethics	Test methods of operation were reviewed and approved by the Commission on Science Standard animal ethics (IACUC22-0046)	Test methods of operation were reviewed and approved by the Commission on Science Standard animal ethics (IACUC22-0045)
There were no known contaminants present in the feed and water expected to interfere with the test data.		

Supplementary Tab. 10 | Input and output of the in situ lignin regeneration treatment method presented in this work.

Input/Output	Quantity	Unit
Step 1: CSBO preparation		
**Input**		
Epoxidized soybean oil（Refined soybean oil is used as an alternative）	310	kg
Tetrabutylammonium iodide	5.15	kg
L-Ascorbic Acid（Ascorbic Acid is used as an alternative）	2.45	kg
CO_2	54.06	kg
Energy	0.066	kWh
**Output**		
CSBO	318	kg
Step 2: APAC Preparation		
**Input**		
Diglycidyl ester of acrylpimaric acid	50	kg
Tetrabutylammonium iodide	0.76	kg
L-Ascorbic Acid（Ascorbic Acid is used as an alternative）	0.36	kg
CO_2	8	kg
Energy	7.91	kWh
**Output**		
APAC	52	kg
Step 3: Paper plastic preparation		
**Input**		

CSBO	310	kg
APAC	50	kg
PEI	140	kg
Paper	500	kg
Energy[a]	62.4	kWh
**Output**		
Paper plastic	1000	kg

[a]The energy includes the electricity for mixing CSBO and APAC with PEI, coating on paper, and curing.

Supplementary Tab. 11 | Density and tensile strength used to normalize the environmental impacts.

Material	Density ($g·cm^{-3}$)	Tensile strength (MPa)
Acrylonitrile butadiene styrene (ABS)	1.100	45.0
Polypropylene (PP)	0.900	31.8
Polyethylene terephthalate (PET)	1.345	60.4
Low-density polyethylene (LDPE)	0.949	32.8
Polybutadiene (PB)	0.910	15.0
Polycarbonate (PC)	1018	65.0
PU rigid foam (PU)	0.200	2.3
Polylactic acid (PLA)	1.250	55.0
Polyvinyl chloride (PVC)	1.360	37.0
Polyvinylfluoride (PVF)	1.470	50.0
Polyvinylidenchloride (PVDC)	1.680	67.0
Poly(methyl methacrylate) (PMMA)	1.190	75.0
Polyamide/glass fiber (PA/GF)	1.340	91.0
ABS/carbon fiber (ABS/CF)	1.265	106.2

Supplementary Tab. 12 | Summary of microplastic characteristic fragments, quantitative ions, standard curves, R^2 and limits of quantification produced by 11 standard plastic products.

Plastic	Characteristic fragment	Quantitative ions (m/z)	Standard curves	R^2	Limits of quantification (LOQ, μg)
PS	Styrene trimer	91	Y=11950370X–247312.1	0.9998	0.02
PE	1-Decene	111	Y=700254.5X–169232.9	0.9992	0.22
PP	2,4-Dimethyl-1-heptene	70	Y=990671.3X–5387.353	0.9966	0.02
PMMA	Methyl methacrylate	100	Y=21752830X–974976.3	0.9973	0.02
PVC	Naphthalene	128	Y=1677993X–399449.8	0.9995	0.06
PC	Bisphenol A	228	Y=2836385X–237842.2	0.9994	0.02
PET	Vinyl benzoate	105	Y=766762.9X–171277.3	0.9970	0.10
PA6	Caprolactam	85	Y=6070242X–1058689	0.9967	0.06
PA66	Cyclopentanone	84	Y=4642579X–724033.6	0.9985	0.06
PLA	DL-Lactide	56	Y=2031387X–630451.4	0.9988	0.10
PBAT	(S,S)-2,3-Butanediol	72	Y=4262826X–295504.4	0.9966	0.10

Supplementary Tab. 13 | Summary of microplastics test results for paper plastics.

Name	Microplastic content in the sample (μg/g)										
	PS	PE	PP	PMMA	PVC	PC	PET	PA6	PA66	PLA	PBAT
Paper plastic	N.D.	N.D.	N.D.	N.D.	N.D.	N.D.	N.D.	N.D.	N.D.	N.D.	N.D.
N.D. indicates below the detection limit or not detected.											

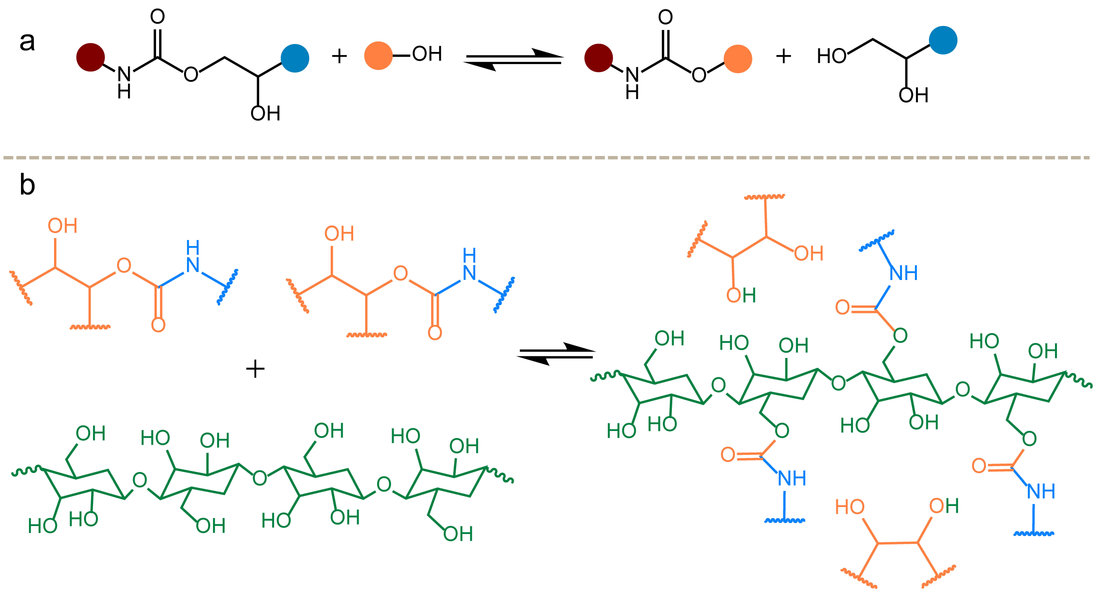

Supplementary Fig. 1 | Schematic diagram of the dynamic carbamate chemistry. a, the bond exchange mechanism of the carbamate bond. b, the schematic diagram of the exchange of NIPU network with cellulose paper.

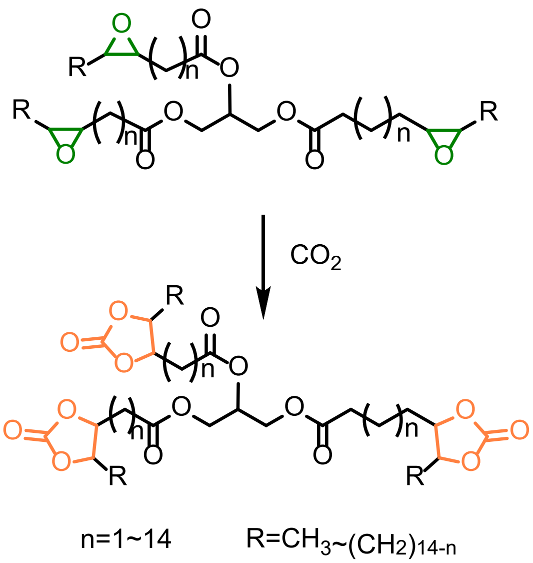

Supplementary Fig. 2 | Synthesis of cyclic-carbonated soybean oil (CSBO).

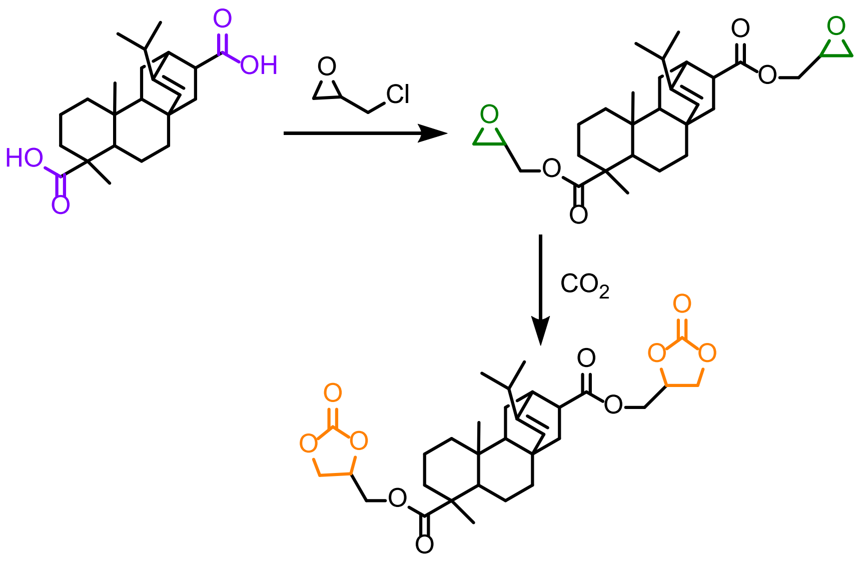

Supplementary Fig. 3 | Synthesis of acrylpimaric acid cyclic carbonate (APAC).

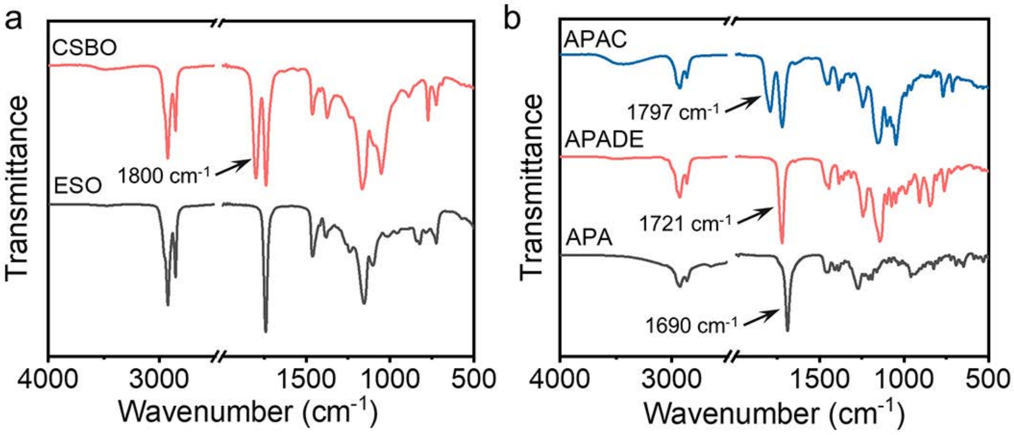

Supplementary Fig. 4 | FTIR spectra of raw materials. a, epoxidized soybean oil (ESO) and cyclic-carbonated soybean oil (CSBO). b, acrylpimaric acid (APA), acrylpimaric acid glycidyl ether (APADE), and acrylpimaric acid cyclic carbonate (APAC).

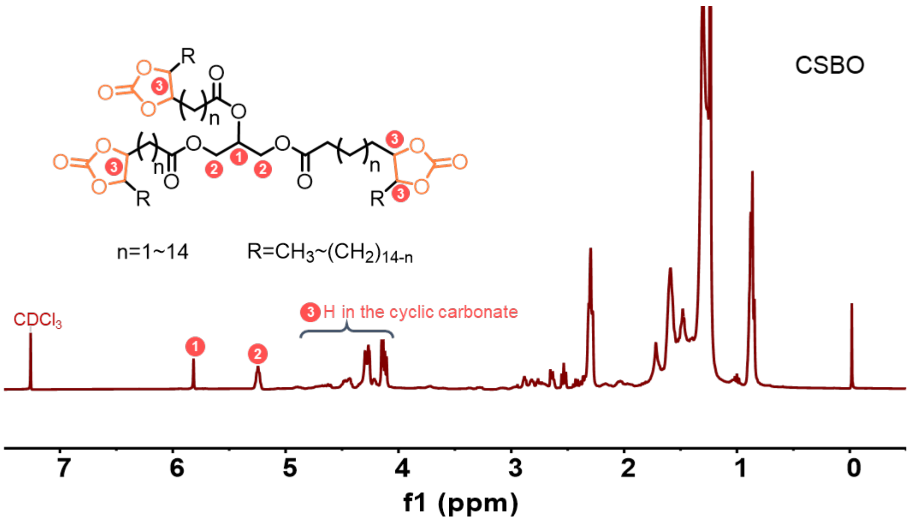

Supplementary Fig. 5 | ^{1}H NMR (solvent: $CDCl_3$, 25 °C, 400 MHz) spectra of the raw material cyclic-carbonated soybean oil (CSBO).

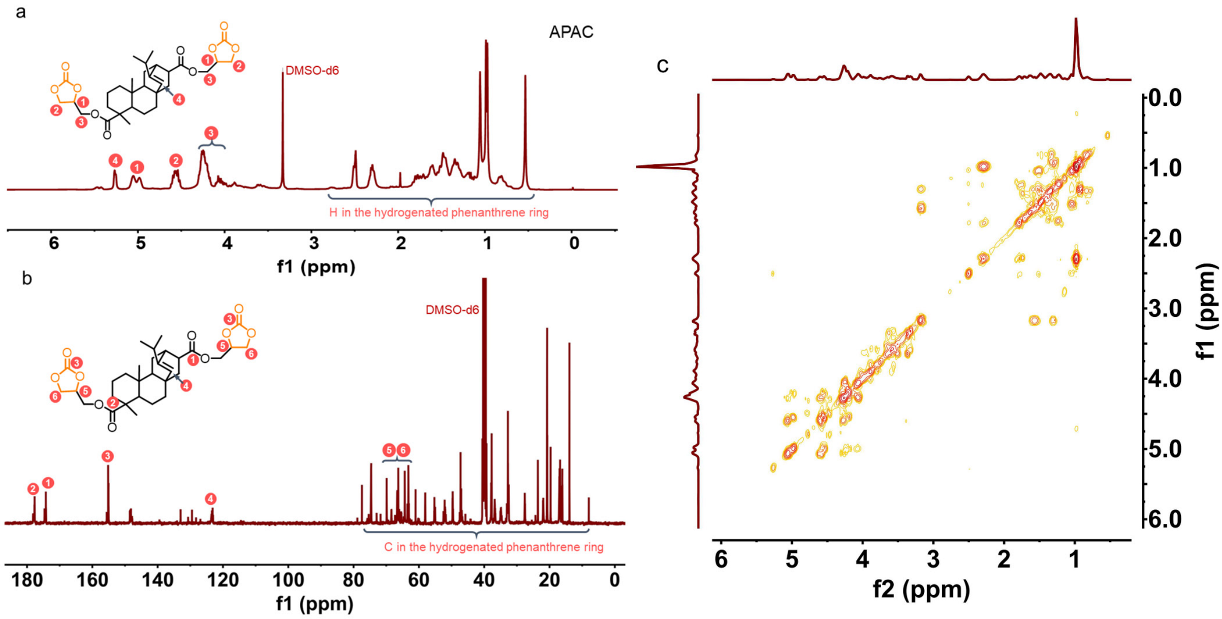

Supplementary Fig. 6 | NMR spectra (solvent: DMSO-d6, 25 °C, 400 MHz) of the raw materials (cyclic carbonate APAC). a, ^{1}H NMR spectrum. b, ^{13}C NMR spectrum. c, 2D NMR (COSY) spectrum.

The chemical shifts belonging to the cyclic carbonate groups appear in the NMR spectra, confirming the successful reaction of the epoxide with CO_2 to form a cyclic carbonate.

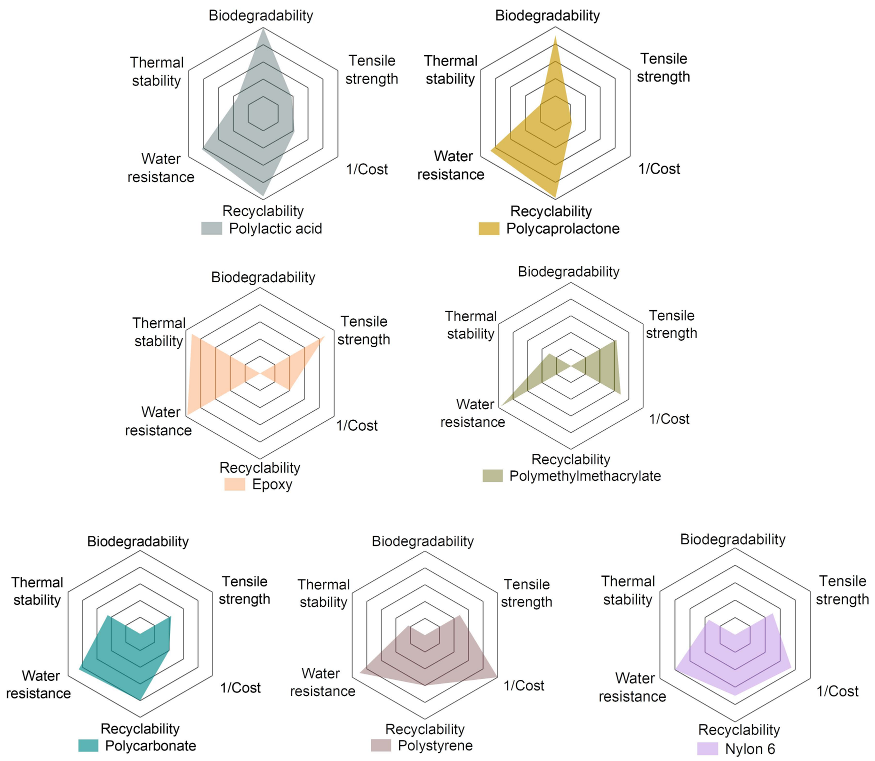

Supplementary Fig. 7 | Radar plots comparing the performance of commercial plastics, in which the results are normalized by the maximum value of each characteristic.

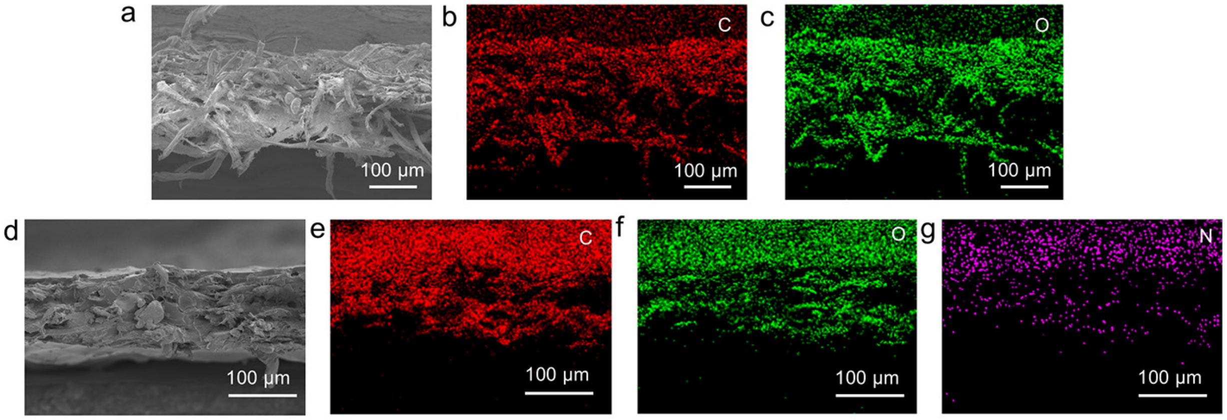

Supplementary Fig. 8 | Image analysis to reveal the morphologies of the fracture cross section in paper and paper plastic. a, cross-sectional SEM image in paper. b, c, EDX mappings of C (b) and O (c) in the fracture cross section. d, cross-sectional SEM image in paper plastic. e–g, EDX mappings of C (e), O (f), and N (g) in the fracture cross section.

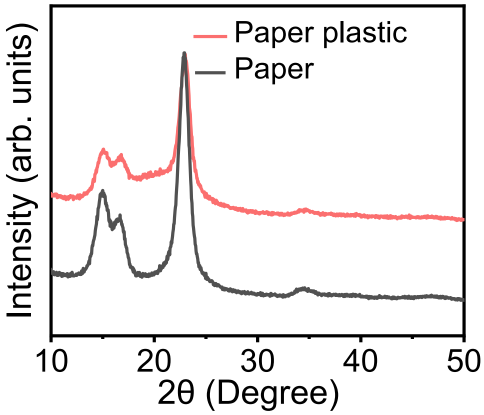

Supplementary Fig. 9 | XRD patterns of paper and paper plastic.

The XRD spectra demonstrate the crystalline structure of paper (cellulose)[3, 4] is retained by the paper plastic, as evidenced by peaks at 2θ of 15.06°, 16.78°, 22.86°, and 34.61° corresponding to the lattice (101), (10ī), (002), and (040), respectively. The results indicate that the incorporation of NIPU into the paper has negligible impact on the crystalline structure.

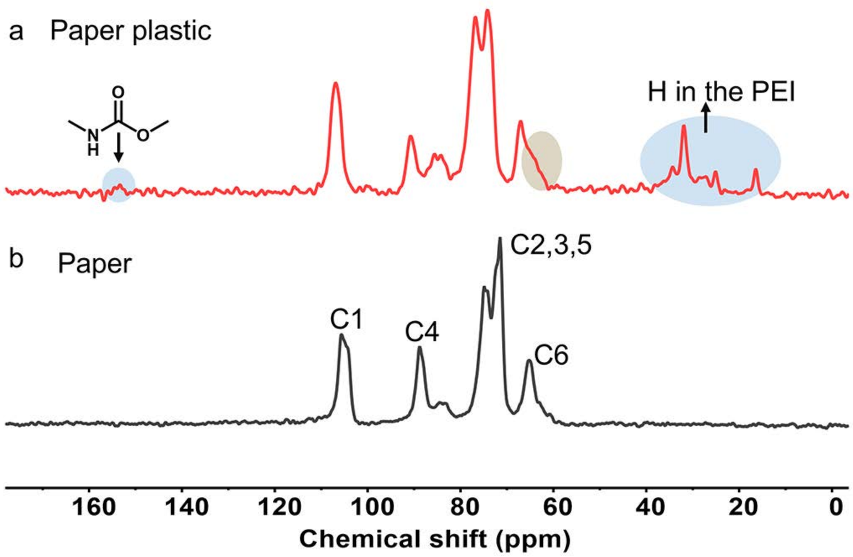

Supplementary Fig. 10 | ^{13}C Solid-state NMR spectra of paper and paper plastic.

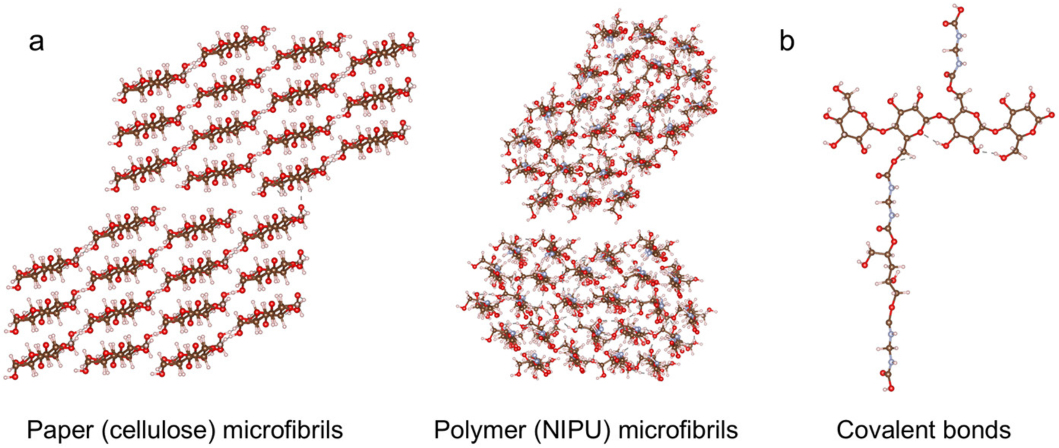

Supplementary Fig. 11 | Structures in all-atom molecular dynamics simulations. a, Atomistic structures of cellulose microfibrils and NIPU microfibrils. b, Atomistic structures of cellulose chains and NIPU chains.

All-atom molecular dynamics simulations. To further investigate the interaction between cellulose microfibrils and NIPU microfibrils and provide atomistic information for subsequent coarse-grained molecular dynamics simulations, we calculated the mechanical behavior of cellulose microfibrils, NIPU microfibrils, and their assembly. The Young's modulus of NIPU microfibrils was estimated as 15 GPa based on all-atom molecular dynamics simulations, while the Young's modulus of cellulose microfibrils was adopted as 20 GPa based on experimental measurements[5–7]. The interchain interactions, encompassing van der Waals and hydrogen-bonding interactions, were found to be relatively weak through simulations of paper microfibril separation[8] (Supplementary Fig. 11a and Supplementary Tab. 2). In addition to these relatively weak interchain interactions, it was observed that cellulose chains could form strong covalent bonds with NIPU chains (Supplementary Fig. 11b). There are relatively weak interchain interactions (van der Waals interactions and hydrogen-bonding interactions) in cellulose microfibrils/cellulose microfibrils, NIPU microfibrils/NIPU microfibrils. However, the interchain interactions in NIPU microfibrils/cellulose microfibrils were dominated by strong covalent bonds, which account for the significantly higher binding energy.

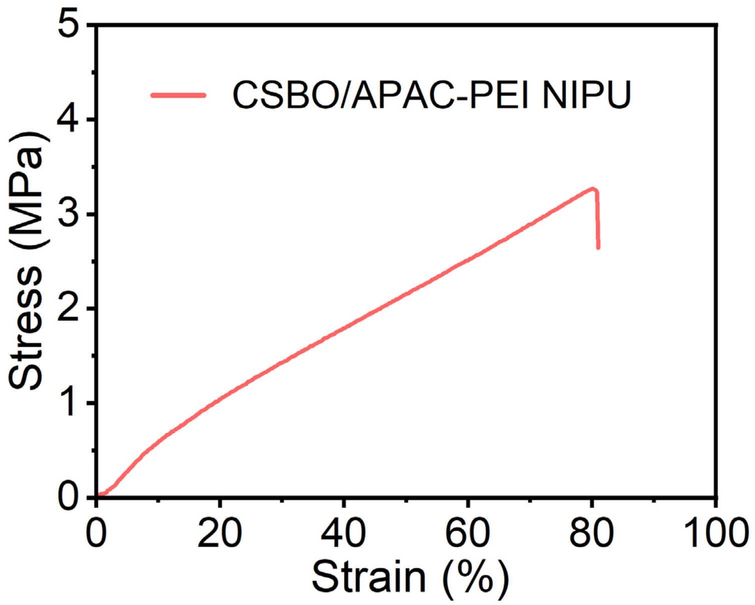

Supplementary Fig. 12 | Tensile stress–strain curve of NIPU (CSBO/APAC–PEI).

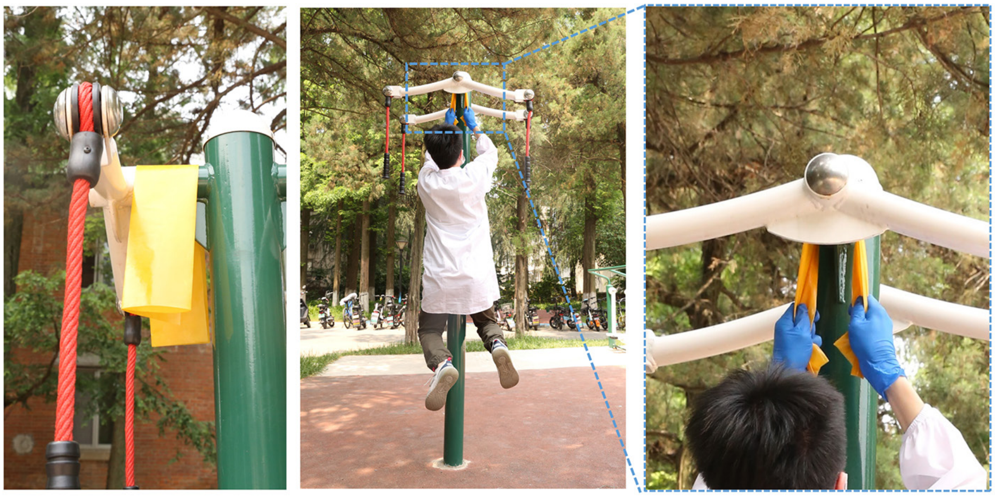

Supplementary Fig. 13 | Paper plastic strip (three layers) with a width of 100 mm and thickness of 0.12 mm can withstand a 65 kg boy.

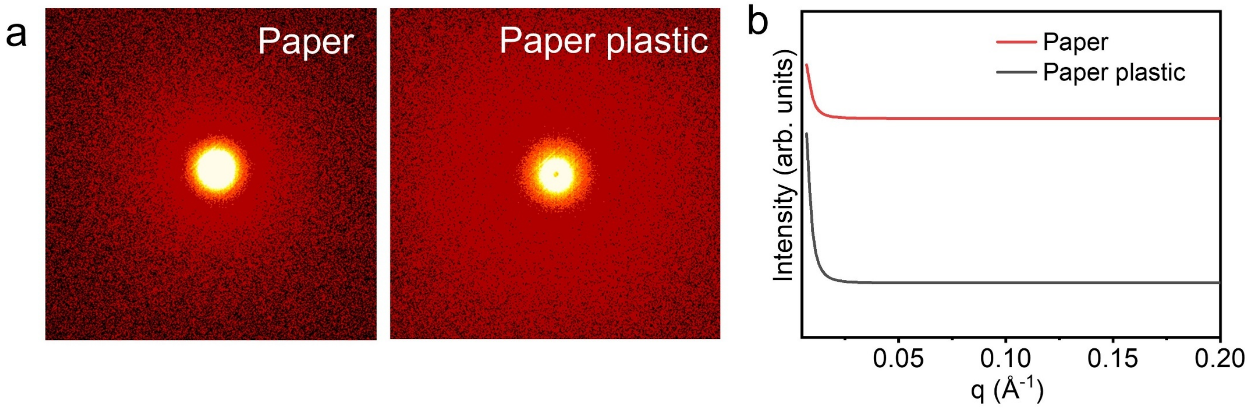

Supplementary Fig. 14 | SAXS tests of paper and paper plastic. a, 2D small angle X-ray scattering (SAXS) patterns of paper and paper plastics. b, SAXS curves of paper and paper plastics. The scattering pattern shows the isotropic nature of the material, with no scattering peaks observed in the paper and paper plastics. This suggests the molecular chains in the material are disordered.

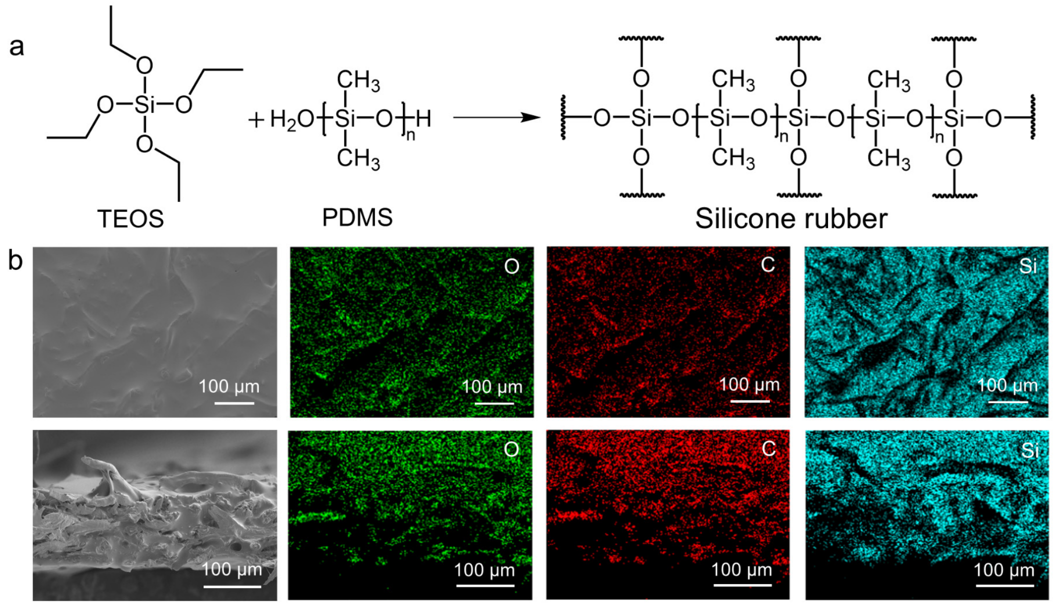

Supplementary Fig. 15 | Micro-morphology images of silicone rubber. a, The synthesis process of silicone rubber. b, The surface and cross-sectional SEM analyses, and the EDS mapping of O, C, Si.

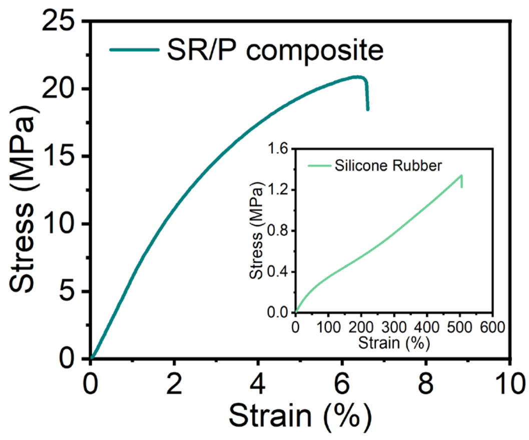

Supplementary Fig. 16 | Tensile stress–strain curve of silicone rubber modified paper (SR/R composite). The inset corresponds to the tensile stress–strain curve of silicone rubber.

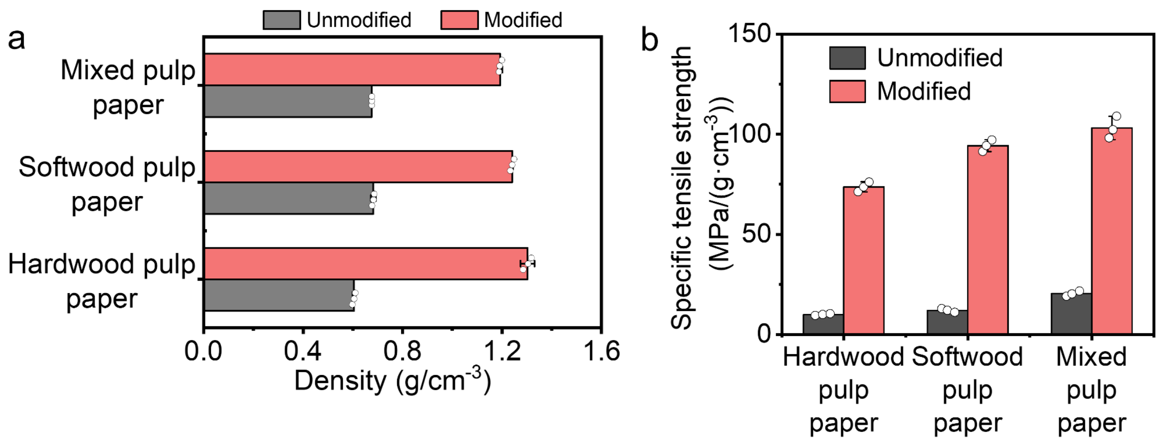

Supplementary Fig. 17 | Density changes and Specific tensile strength of different paper types before and after preparation into paper plastics. Data in a and b are reported as their means ± SDs from n = 3 independent samples.

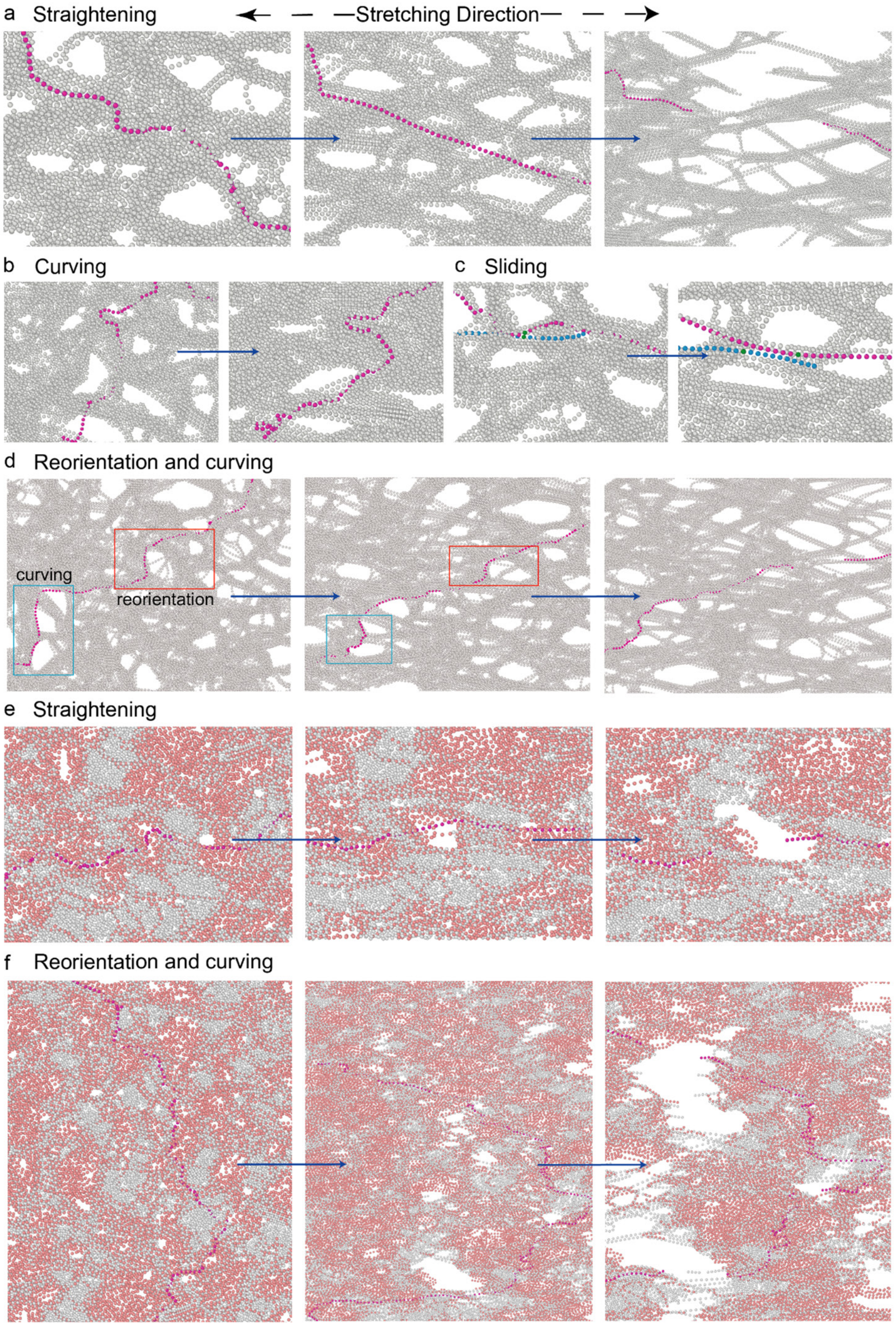

Supplementary Fig. 18 | Moving patterns of microfibrils under uniaxial stretching. a–d, In cellulose paper models, except for straightening, reorientation, and curving, the sliding pattern is also a common phenomenon. e–f, In paper plastic models, little sliding patterns between cellulose microfibrils and polymer microfibrils can be found, due to the strong bonding interactions. The chains with grey beads represent paper (cellulose) microfibrils, and the pink ones represent polymer microfibrils.

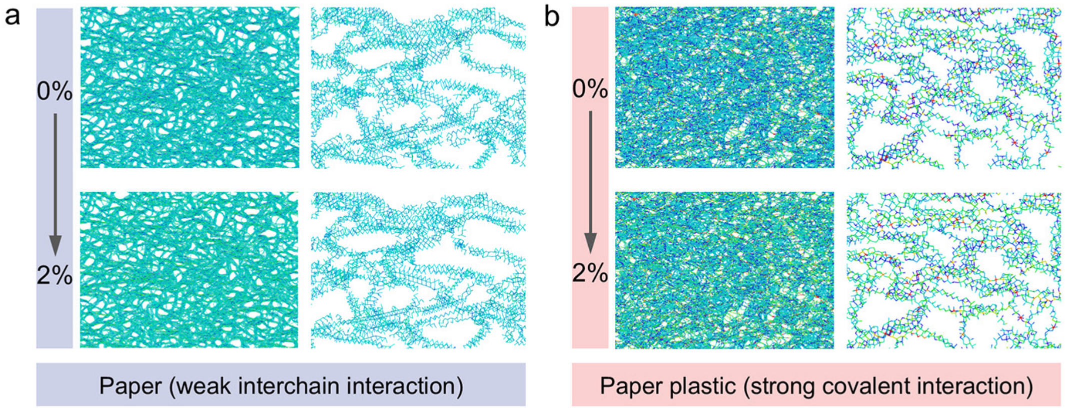

Supplementary Fig. 19 | Load-transfer during stretching. a, Color-coded two-dimensional maps of stresses carried by cellulose paper at a strain of 2%. b, Color-coded two-dimensional maps of stresses carried by paper plastic at a strain of 2%.

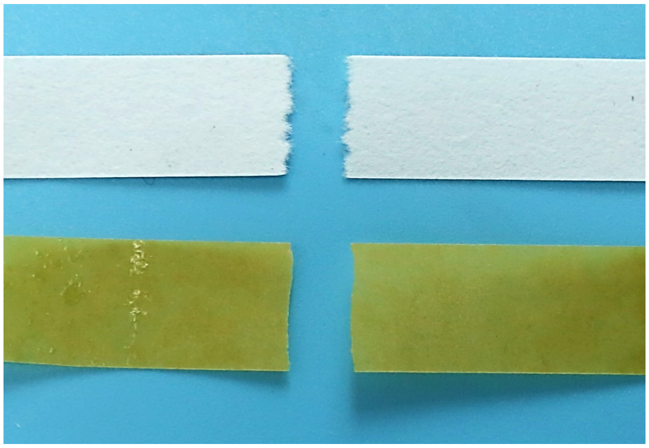

Supplementary Fig. 20 | Images of the fracture appearance of the paper and paper plastic after tensile tests.

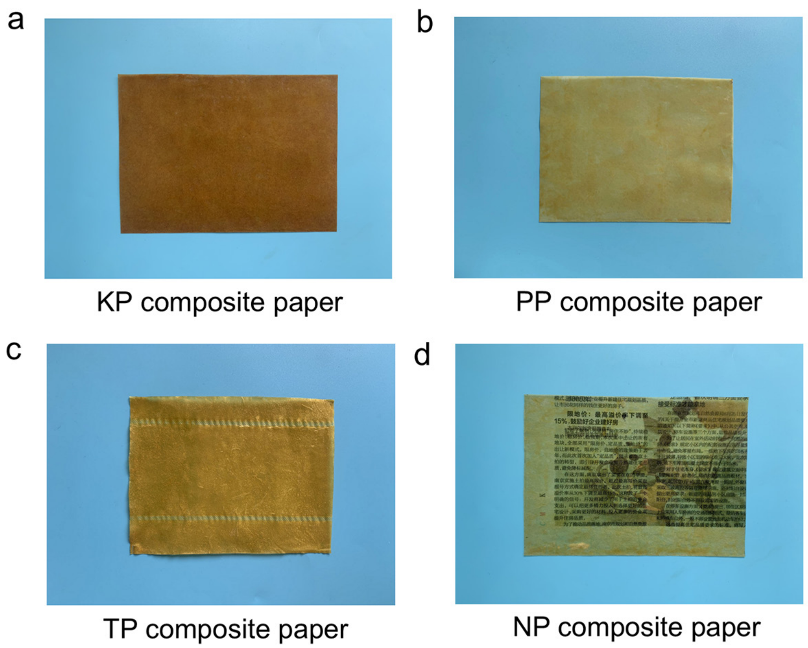

Supplementary Fig. 21 | The photographs of different types of paper prepared into paper plastic. a, Modified kraft paper (KP). b, Modified printer paper (PP). c, Modified tissue paper (TP). d, Modified newspaper (NP).

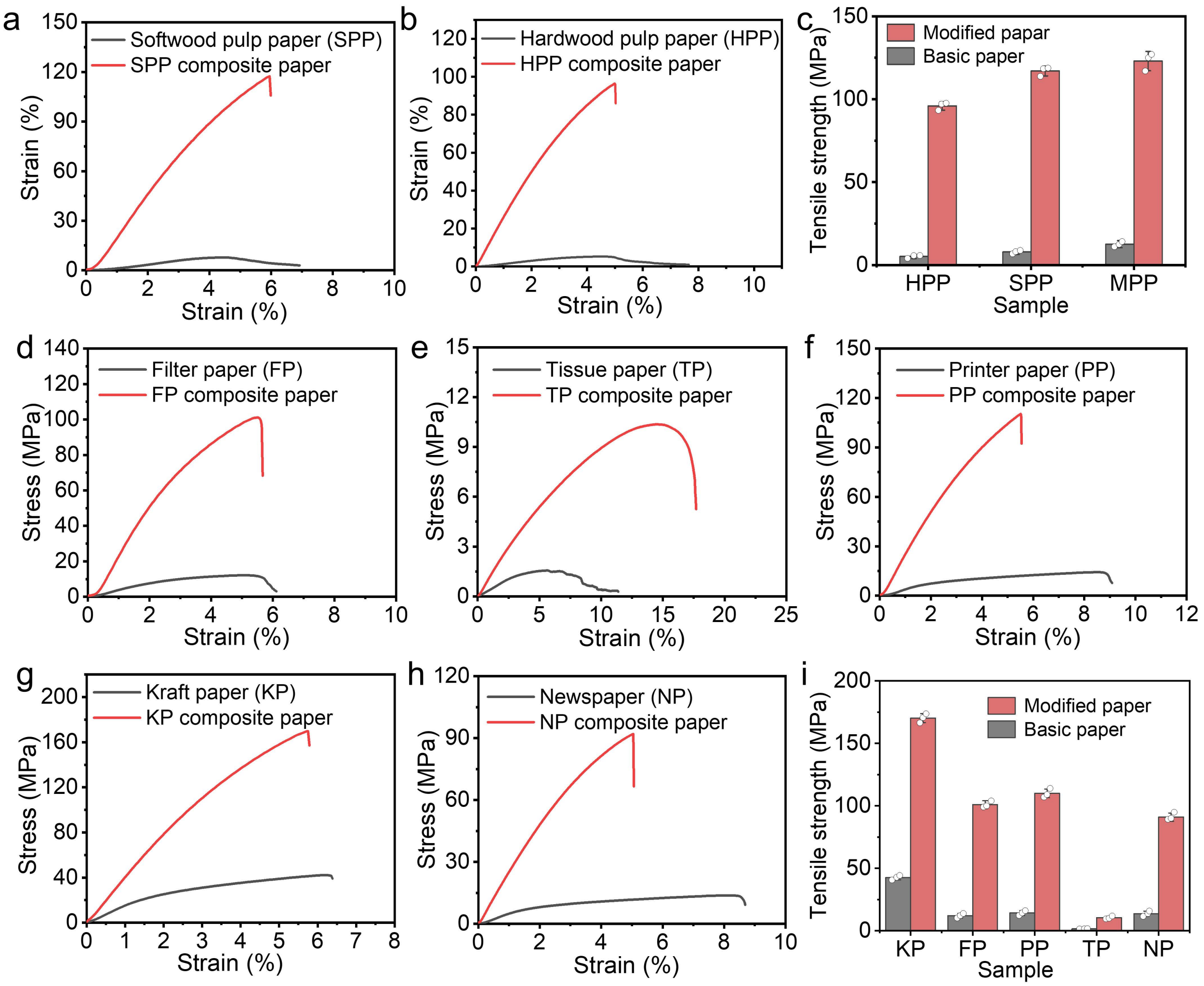

Supplementary Fig. 22 | Mechanical properties of paper plastics before and after modification. a, Softwood pulp paper. b, Hardwood pulp paper. c, Comparison of tensile strength. d, Filter paper. e, Tissue paper. f, Printer paper. g, Kraft paper. h, Newspaper. i, Comparison of tensile strength. Data in c and i are reported as their means ± SDs from n = 3 independent samples.

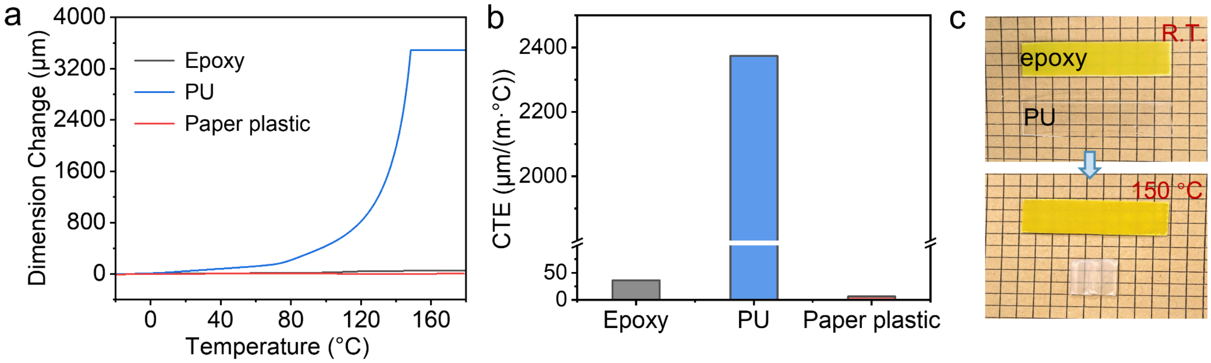

Supplementary Fig. 23 | Thermal stability of epoxy and polyurethanes (PU). a, Thermal expansion. b, Coefficient of thermal expansion (CTE). c, Dimensional stability experiment at 27 °C (R.T.) and 150 °C.

We purchased commercial epoxy resins and polyurethanes (PU) from Jiangsu Zhongtian Rubber & Plastic Company and performed thermal expansion tests using a thermomechanical analyzer (TMA Q400). The coefficient of thermal expansion (CTE) of the materials was also recorded from –20 °C to 180 °C. In Supplementary Fig. 23, the epoxy exhibits good dimensional stability and a low coefficient of thermal expansion (CTE, 36 μm/(m·°C)) as the temperature changes. Paper plastic has similar thermal dimensional stability with epoxy resin due to the stability of the paper structure. PU exhibits less ideal thermal stability, which is manifested in significant dimensional changes during temperature changes and fractures in the middle of the process. After heating epoxy resin and PU in an oven at 150 °C for 20 min (Supplementary Fig. 23c), the size of epoxy did not change visibly due to its excellent thermal stability, while PU showed curling deformation.

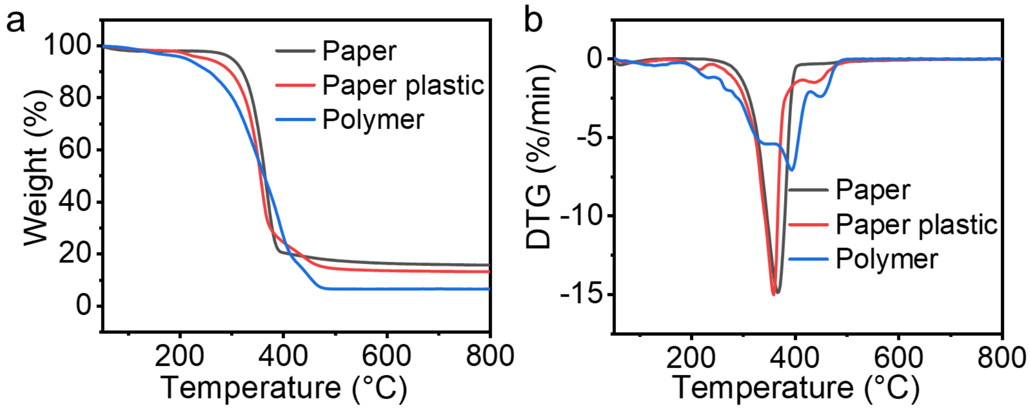

Supplementary Fig. 24 | Thermal stability. a, TGA and b, DTG curves of paper, polymer (NIPU), and paper plastic.

Thermogravimetric analysis (TGA) was used to examine the thermal decomposition behavior of the paper plastic. As shown in Supplementary Fig. 24a, the initial weight loss temperature ($T_{5\%}$) of the paper plastic is 252 °C, an intermediate value between those of NIPU and cellulose paper. Simultaneously, the maximum thermal weight loss of paper plastic occurred at a temperature of 357 °C, which is only slightly lower than that of pure paper (366 °C) (Supplementary Fig. 24b).

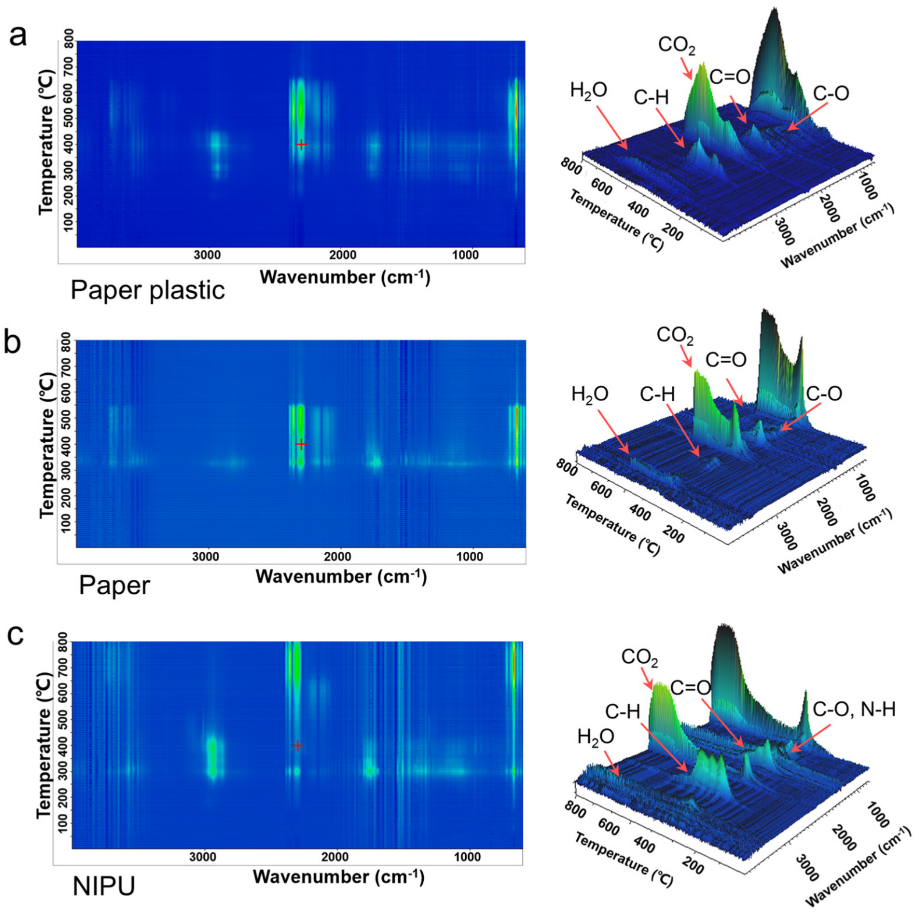

Supplementary Fig. 25 | 2D and 3D TG–FTIR spectra, as a function of FTIR wavenumber and TG temperature. a, Paper plastic. b, Paper. c, Polymer (NIPU).

To have a more mechanistic understanding of the thermal decomposition process, TG–FTIR test was performed to record the real-time correlation between pyrolysis gas type and concentration with temperature[9] (Supplementary Fig. 25). Supplementary Fig. 25 presents the temperature-dependent concentration distribution of decomposition products. As seen, the concentration of C–H species and CO_2 for paper plastic is significantly higher than that for cellulose paper and lower than that in NIPU due to the incorporation of alkane chain segments. Moreover, because of the exceptional thermal stability exhibited by paper, the temperatures of forming C–H species and CO_2 gas in paper plastic are delayed compared to that of NIPU, which confirms the enhanced thermal stability of paper plastic.

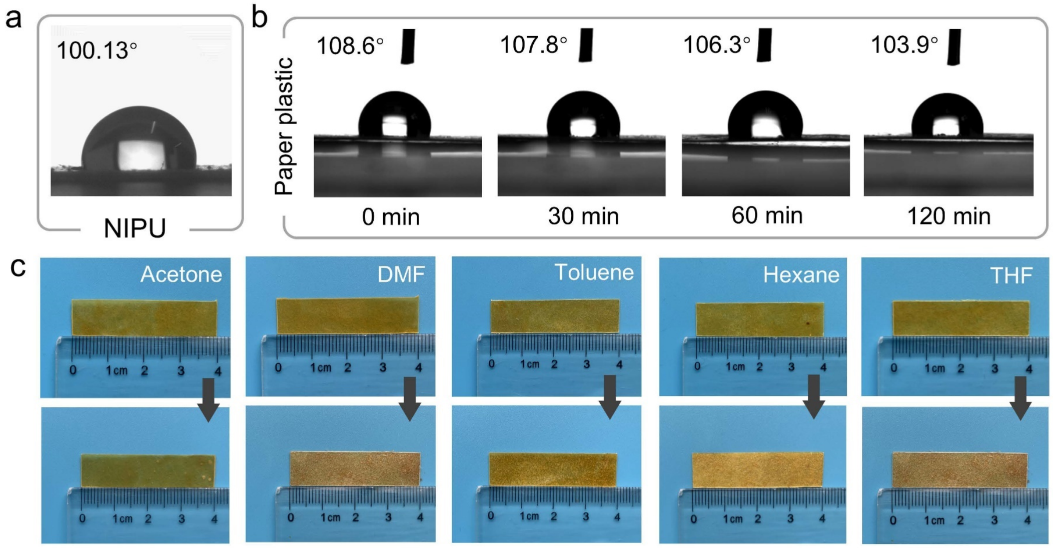

Supplementary Fig. 26 | Analysis of water and solvent resistance. a, Water contact angle of NIPU. b, Change in water contact angle of paper plastic over time. c, Images of the paper plastic after being immersed in different organic solvents for 7 days.

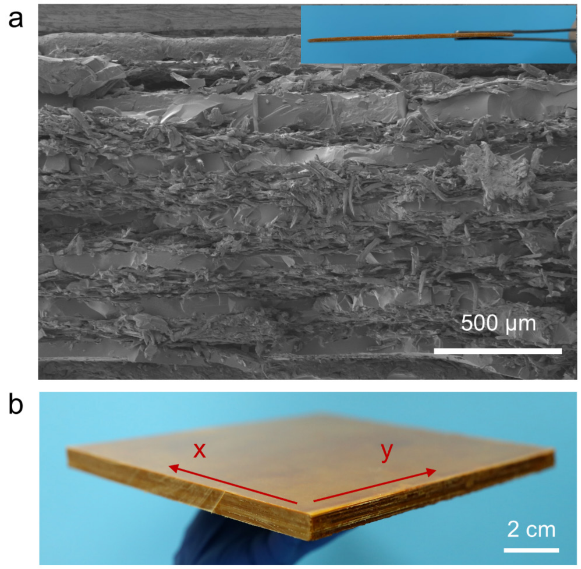

Supplementary Fig. 27 | The morphology of paper plastic board. a, SEM image analysis of the closely cross-linked cross-sections of the multi-layer (6 layers) paper plastic after hot-pressing. The inset corresponds to the multi-layer (6 layers) paper plastic. b, Multiple paper plastic boards with a thickness of about 1 cm.

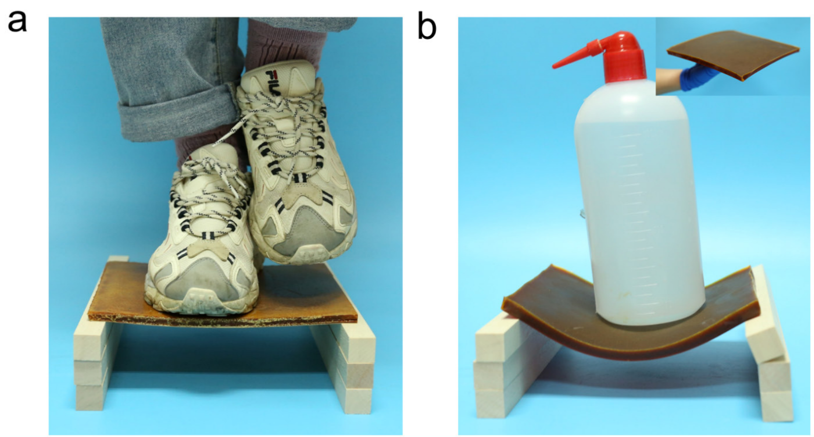

Supplementary Fig. 28 | Comparative images of heavy loads. a, paper plastic. b, NIPU.

Supplementary Fig. 28a demonstrates the load-bearing capacity of the paper plastic board, which showing that it can support a weight of 60 kg. In contrast, Supplementary Fig. 28b illustrates that the NIPU board with size polymer cannot even withstand the weight of hold a 1 kg water bottle.

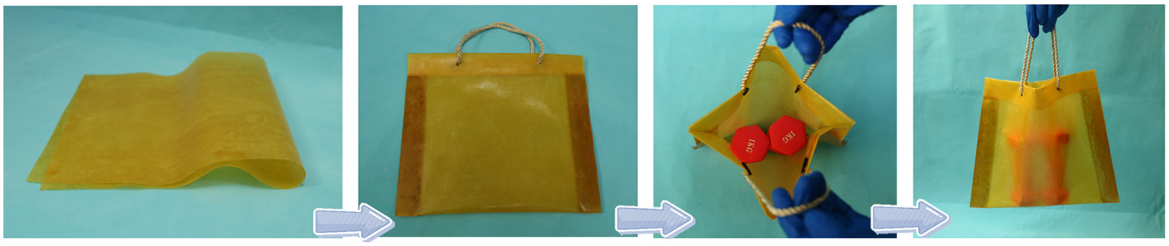

Supplementary Fig. 29 | Schematic illustration of the bag preparation process using the paper plastic.

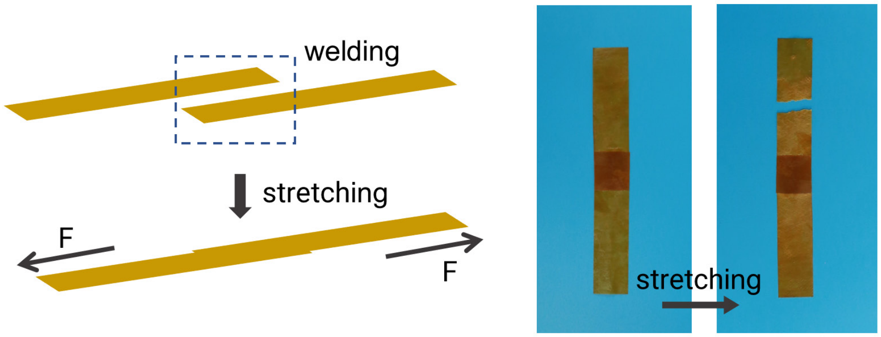

Supplementary Fig. 30 | The schematic diagram of the welding process of two plastic strips and the images after stretching of the welding sample.

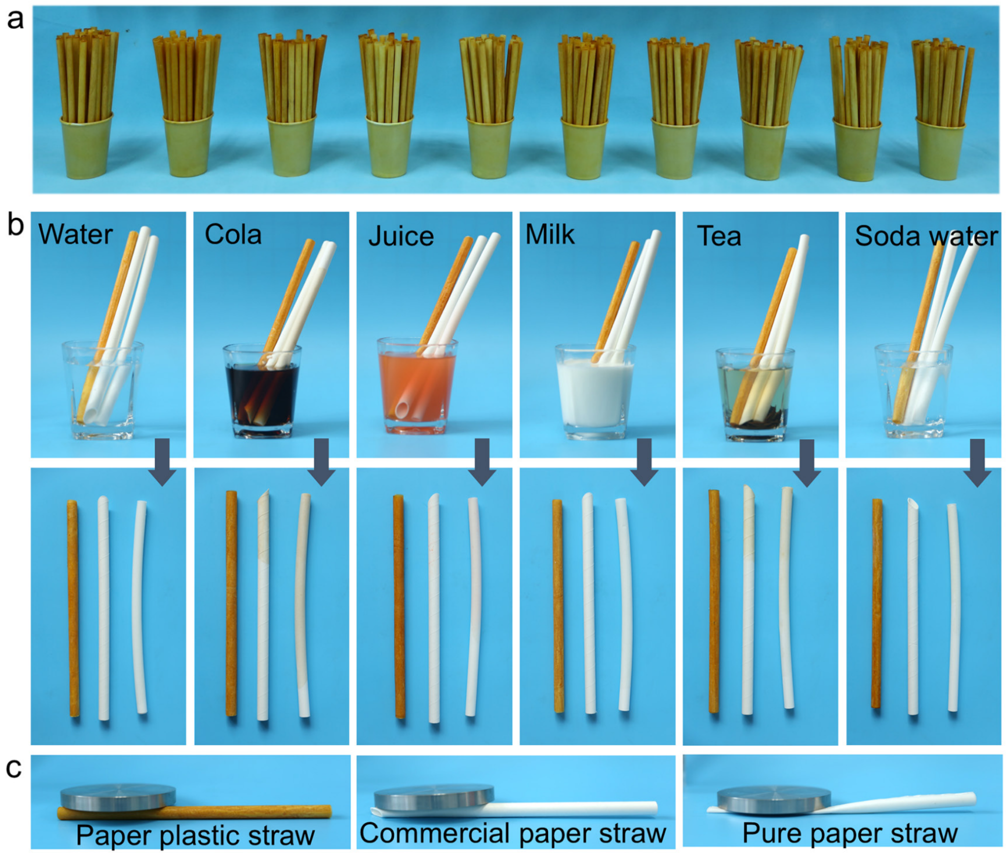

Supplementary Fig. 31 | The products prepared by paper plastic and the solution resistance of the products. a, A large number of cups and straws are made of paper plastic. b, Solution immersing test (water, cola, juice, milk, tea, soda water) for paper plastic, paper, and commercial straw. c, Physical images of the straw being pressed by a heavy object after soaking in water for 6 hours.

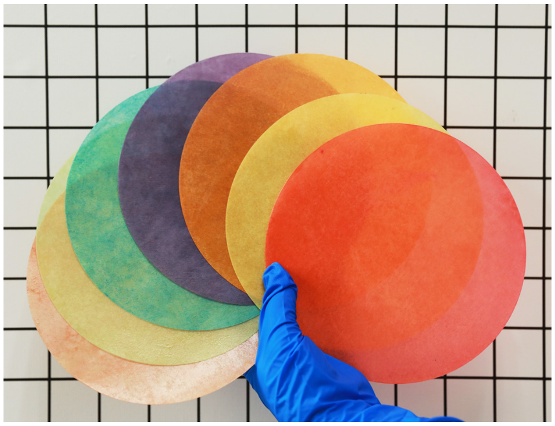

Supplementary Fig. 32 | Paper plastics stained with dye in different colors.

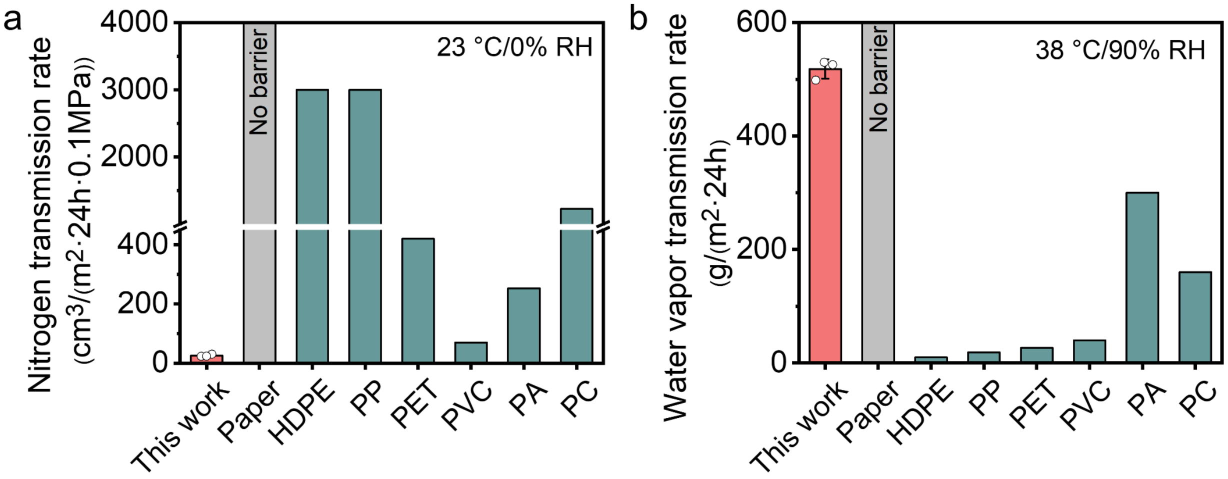

Supplementary Fig. 33 | The barrier properties of paper plastic and commercial plastics. a, The nitrogen transmission rate at 23 °C/90% RH. b, The water vapor transmission rate at 38 °C/90% RH. Data of our work in a and b are reported as their means ± SDs from n = 3 independent samples.

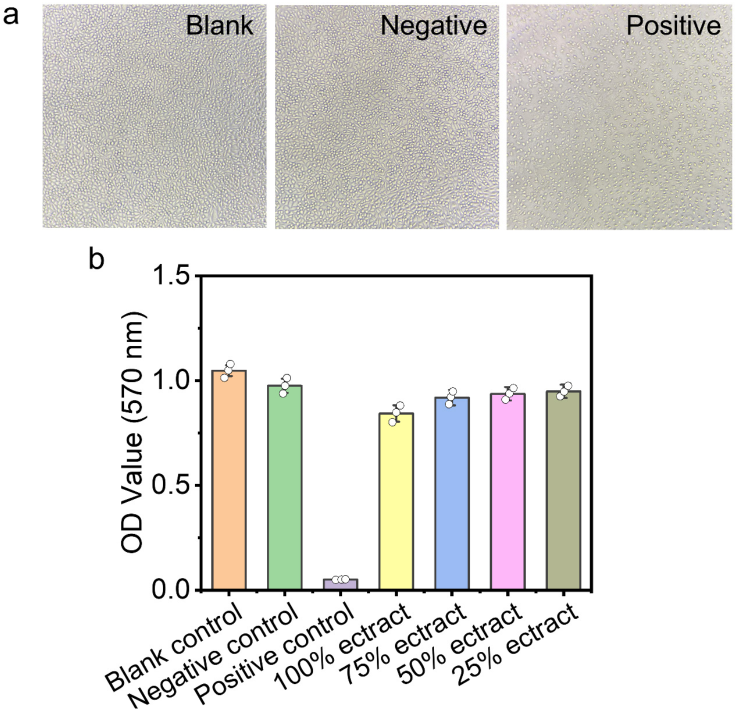

Supplementary Fig. 34 | In vitro cytotoxicity test of paper plastic. a, Cell morphology of positive control, negative control, and blank control. b, OD value of 25%, 50%, 75%, 100% sample extracts, positive control, negative control, and blank control. Data in d are reported as their means ± SDs from n = 3 independent samples.

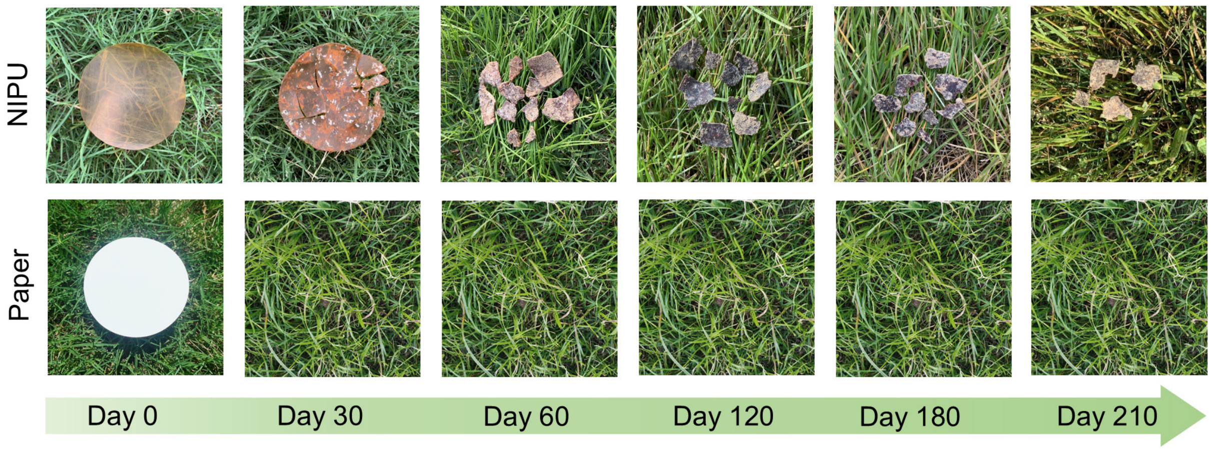

Supplementary Fig. 35 | Degradation process of paper and NIPU buried in soil (Nanjing).

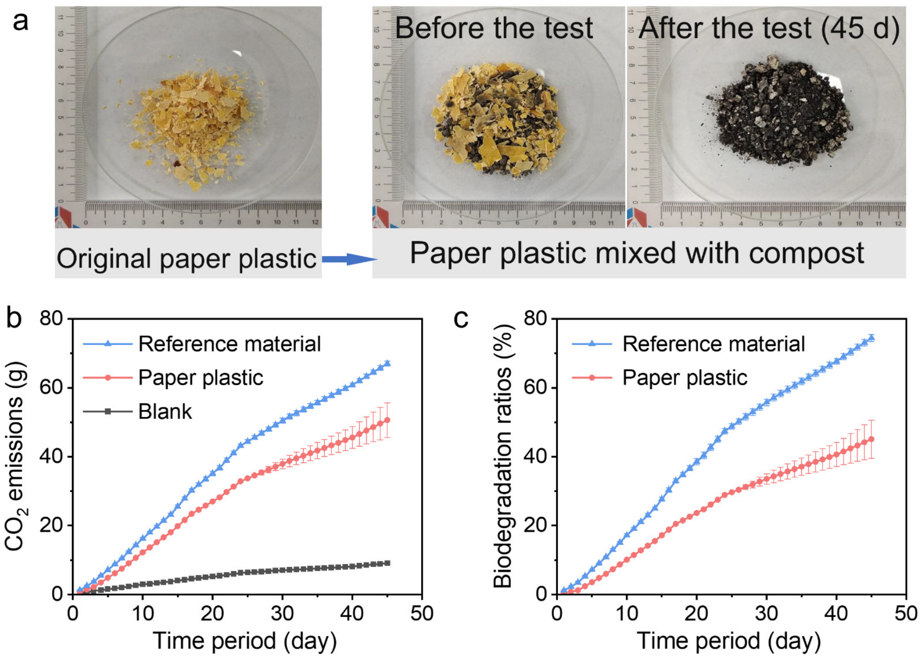

Supplementary Fig. 36 | Determination of the ultimate aerobic biodegradability of paper plastics under controlled composting conditions. a, Physical diagram of paper plastic compost before and after degradation. b, CO_2 emission curve during degradation. c, Biodegradability curve during degradation. Data in b and c are reported as their means ± SDs from n = 3 independent samples.

The amount of CO_2 emitted gradually increases over time as paper plastics are degraded (Supplementary Fig. 36). Based on the cumulative amount of CO_2 released, the biodegradation percentage of paper plastic was 45% ± 5.5% at 45 days, and the biodegradation percentage of the reference material was 74.4% ± 1.0% (> 70%), indicating that the experimental results were reliable and valid. The relative biodegradation rate of paper plastic compared with the reference material is 61%, which also indicates that paper plastic has obvious biodegradability.

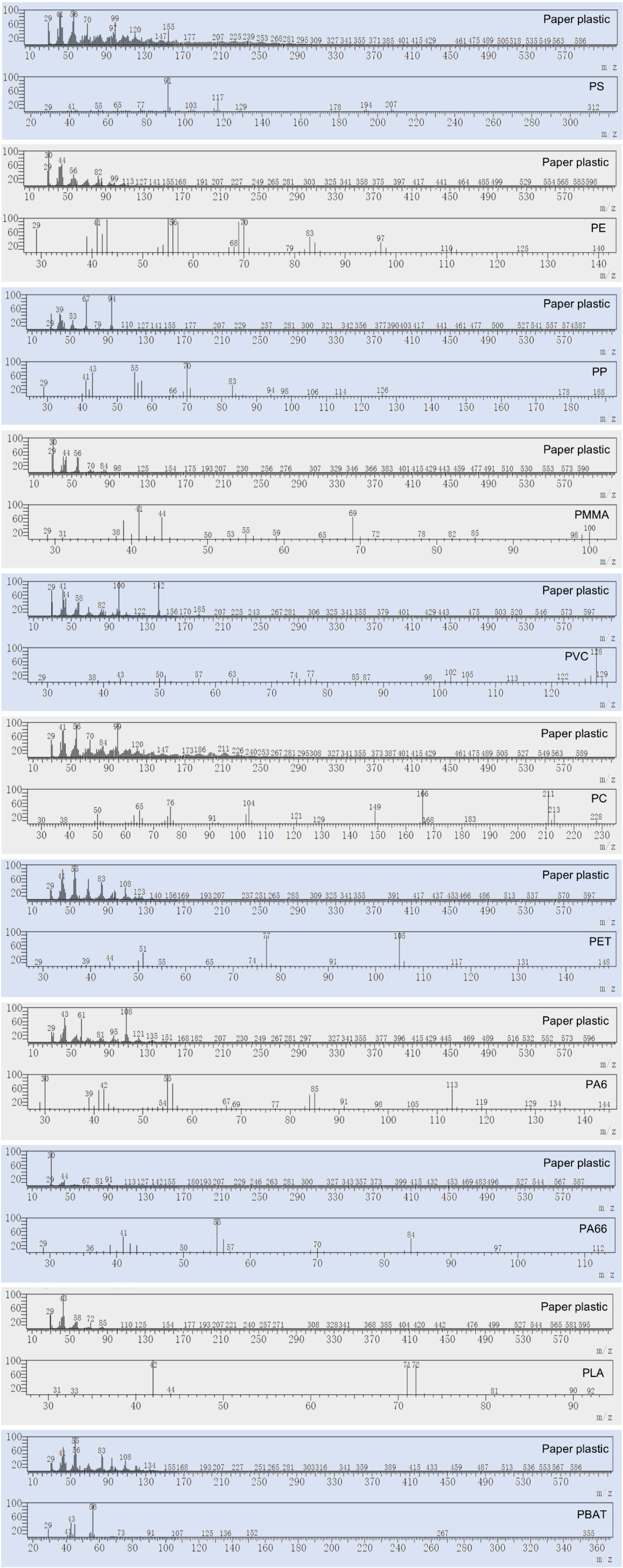
Paper plastic
PS
Paper plastic
PE
Paper plastic
PP
Paper plastic
PMMA
Paper plastic
PVC
Paper plastic
PC
Paper plastic
PET
Paper plastic
PA6
Paper plastic
PA66
Paper plastic
PLA
Paper plastic
PBAT
m/z

Supplementary Fig. 37 | Exploring whether paper plastics produce microplastics. Mass spectra of the pyrolyzed paper plastic compared to the standard plastics.

The characteristic fragments, quantitative ions, standard curves, R^2, and limits of quantification of microplastics produced by 11 standard plastic products are summarized in the Supplementary Tab. 12. From the comparison of gas chromatography and mass spectrometry data, it can be seen that 11 common microplastics were not detected in paper plastics (Supplementary Tab. 13). Therefore, to a certain extent, it can be considered that paper plastics are biodegraded during the degradation process, and there is no obvious production of microplastics (Supplementary Fig. 37).

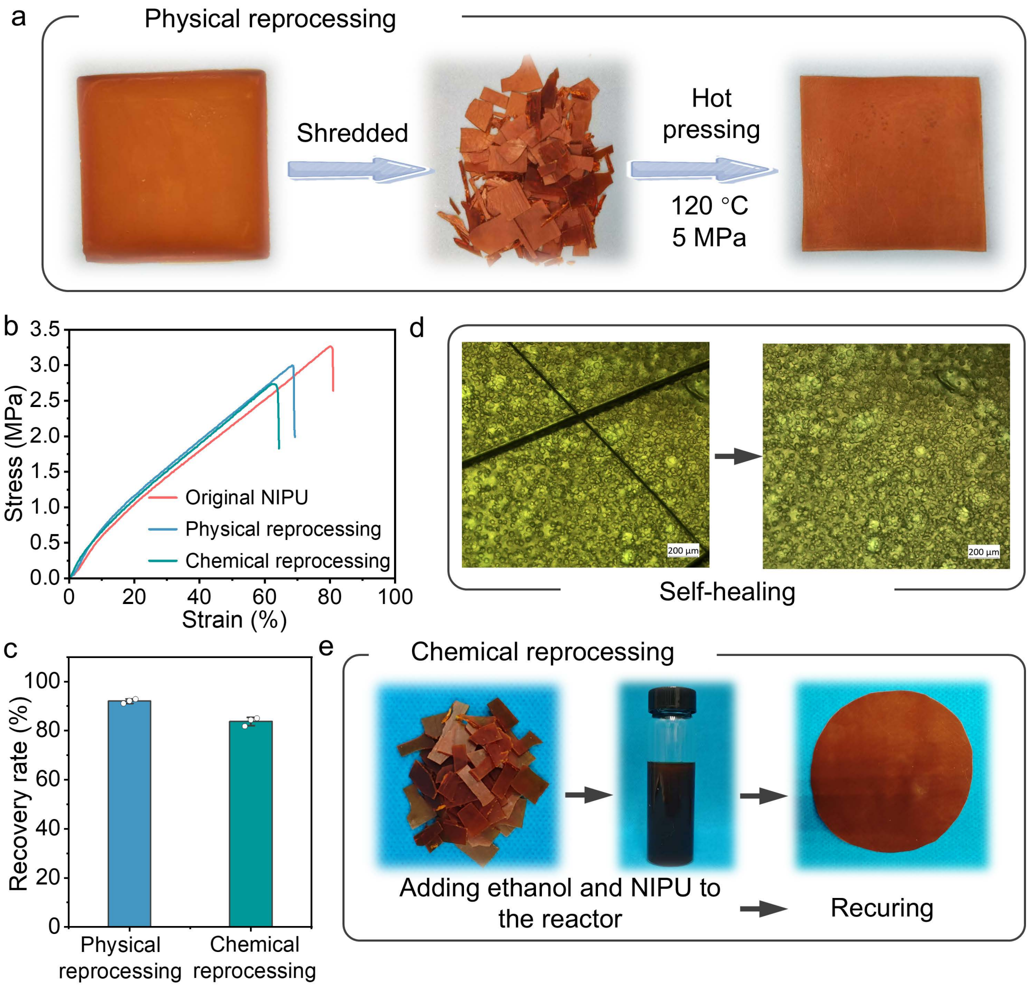

Supplementary Fig. 38 | Recyclability Test of Pure Polymer NIPU. a, Physical Hot Pressing. b, The stress–strain curves of original NIPU and recycled NIPUs. c, The recovery rates of recycled NIPUs with different methods. Error bars in c indicate ± SDs from 3 measurements. d, Self-healing experiments of surface scratches. e, Chemical degradation.

The recovery experiments of the pure polymer in the paper plastic (NIPU) were provided to demonstrate dynamic carbamate chemistry more clearly. The physical hot pressing and chemical degradation are used to explore the dynamic properties. The carbamate bond containing β–OH in the NIPU structure undergoes a dynamic transcarbamoylation reaction after being stimulated by the external temperature, which in turn rearranges the network structure (Supplementary Fig. 1). Therefore, we put the shredded NIPU under a hot press machine and carry out the hot pressing

reprocessing process at a temperature and pressure of 120 °C and 5 MPa. Since the carbamate bond in the structure is bonded with the hydroxyl group, which promotes the network regeneration at the fracture of the material, the material can be effectively cross-linked after 2 hours of hot pressing (Supplementary Fig. 38a). Meanwhile, the mechanical properties of the reprocessed NIPU are tested, which can be restored to more than 90% (Supplementary Fig. 38b and 38c) of the original strength after hot pressing. The scratches on the surface of NIPU can also be self-healing after heat treatment. In addition, the ethanol is used as a solvent to promote the depolymerization of the NIPU polymer network after breaking through the dynamic exchange of hydroxyl groups and carbamate bonds, so that NIPU can be chemical degraded. After collecting the degraded mixed solution system, after rotary evaporation to remove the excess alcohol, the mixture was poured into the Teflon mold and solidified again at 120 °C to prepare NIPU (Supplementary Fig. 38e). The tensile strength of NIPU obtained after re-curing can be restored to 84% (Supplementary Fig. 38b and 38c) of the initial value, which also confirms the dynamics of the network structure. In addition, the recovery rate of chemical degradation is slightly lower than that of the recycled sample after physical hot pressing, which may be due to the fact that some of the small molecule ethanol is still attached to the polymer network, resulting in a slightly reduced degree of cross-linking and a slightly lower recovery rate of mechanical properties than physical recovery.

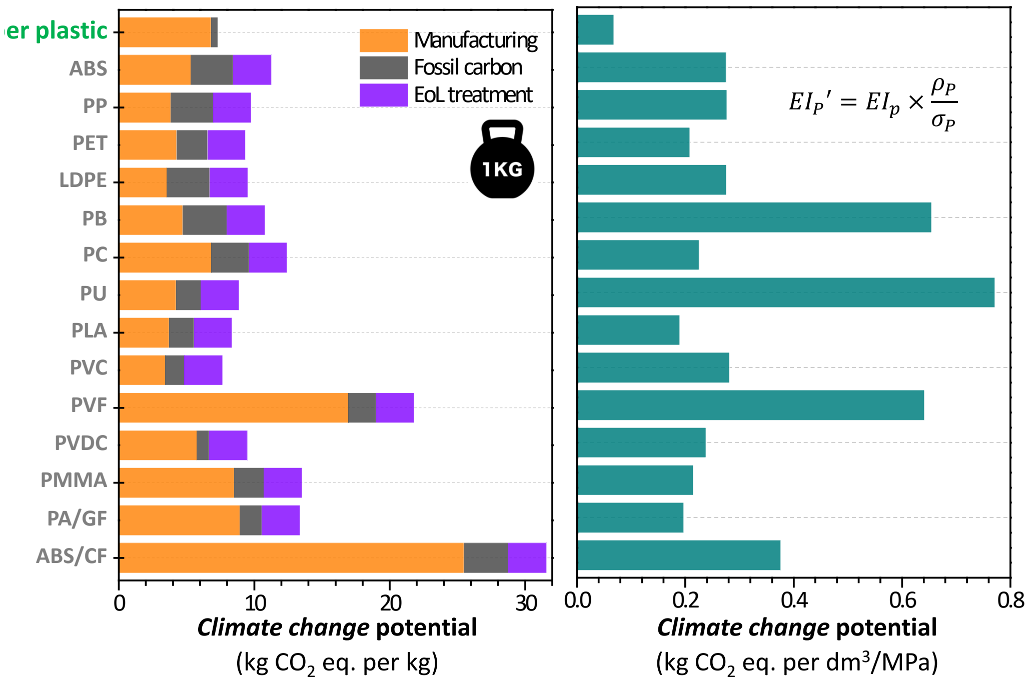

Supplementary Fig. 39 | Comparison of the *climate change* potential of paper plastic to a variety of benchmark plastic films and composite materials according to life cycle assessment (LCA). Left: climate change per 1 kg of material. Right: climate change normalized to tensile strength.

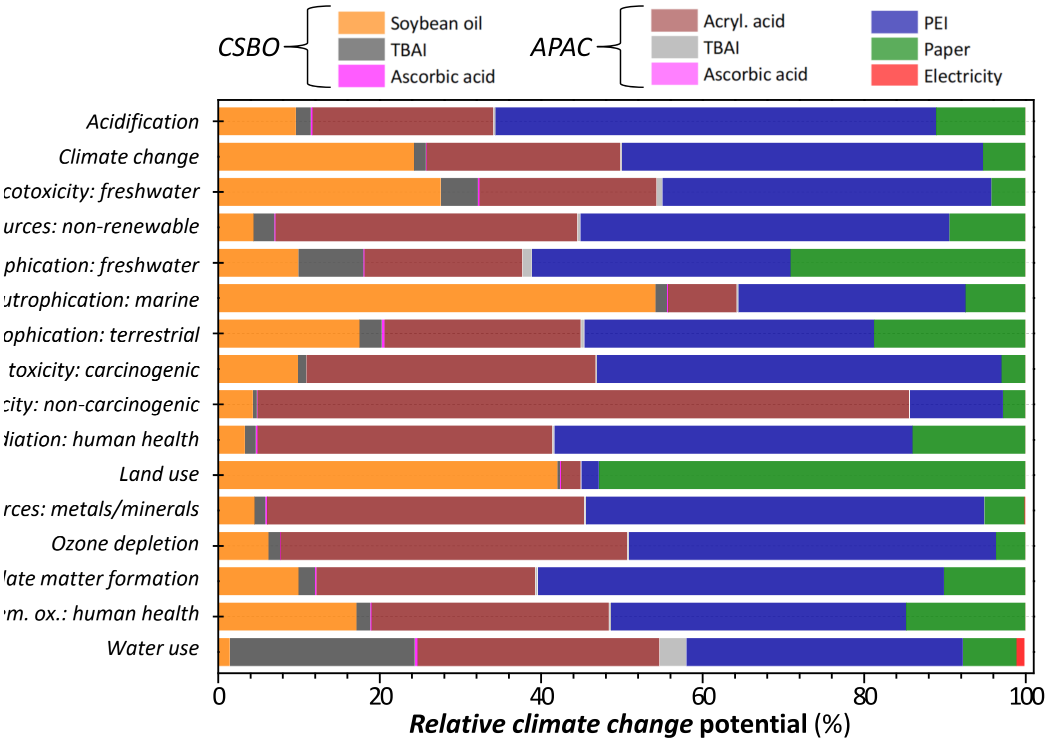

Supplementary Fig. 40 | Environmental impact contribution of each unit in paper plastic production.

Supplementary References

1. Q. Xia, C. Chen, Y. Yao, J. Li, S. He, Y. Zhou, T. Li, X. Pan, Y. Yao, L. Hu, A strong, biodegradable and recyclable lignocellulosic bioplastic. *Nat. Sustain.* **4**, 627-635 (2021).
2. Q. F. Guan, H. B. Yang, Z. M. Han, Z. C. Ling, S. H. Yu, An all-natural bioinspired structural material for plastic replacement. *Nat. Commun.* **11**, 5401 (2020).
3. X. Yang, S. Wang, X. Liu, Z. Huang, X. Huang, X. Xu, H. Liu, D. Wang, S. Shang, Preparation of non-isocyanate polyurethanes from epoxy soybean oil: dual dynamic networks to realize self-healing and reprocessing under mild conditions. *Green Chem.* **23**, 6349-6355 (2021).
4. N. Fanjul-Mosteirin, R. Aguirresarobe, N. Sadaba, A. Larranaga, E. Marin, J. Martin, N.

Ramos-Gomez, M. C. Arno, H. Sardon, A. P. Dove, Crystallization-Induced Gelling as a Method to 4D Print Low-Water-Content Non-isocyanate Polyurethane Hydrogels. *Chem. Mater.* **33**, 7194-7202 (2021).

5. R. Quesada Cabrera, F. Meersman, P. F. McMillan, V. Dmitriev, Nanomechanical and Structural Properties of Native Cellulose Under Compressive Stress. *Biomacromolecules* **12**, 2178-2183 (2011).
6. S. J. Eichhorn, R. J. Young, The Young's modulus of a microcrystalline cellulose. *Cellulose* **8**, 197-207 (2001).
7. Zhang, Y. *et al.* Molecular insights into the complex mechanics of plant epidermal cell walls. *Science* **372**, 706–711 (2021).
8. Oehme, D. P. *et al.* Gaining insight into cell wall cellulose macrofibril organisation by simulating microfibril adsorption. *Cellulose* **22**, 3501–3520 (2015).
9. Wang, D. C. *et al.* Confined Chemical Transitions for Direct Extraction of Conductive Cellulose Nanofibers with Graphitized Carbon Shell at Low Temperature and Pressure. *J. Am. Chem. Soc.* **143**, 11620–11630 (2021).
