# Supplementary material for: Rapidly making biodegradable and recyclable paper plastic based on microwave radiation driven dynamic carbamate chemistry
Source: Nat Commun. 2025 Jul 15;16:6523. doi: 10.1038/s41467-025-61722-0 (PMC12264110; doi:10.1038/s41467-025-61722-0)
Supplement: Supplementary file 2 — Reporting Summary [file 41467_2025_61722_MOESM2_ESM.pdf]

nature portfolio

Corresponding author(s): Chaoji Chen, Xu Xu, He Liu, Enlai Gao

Last updated by author(s): Jun 26, 2025

Reporting Summary

Nature Portfolio wishes to improve the reproducibility of the work that we publish. This form provides structure for consistency and transparency in reporting. For further information on Nature Portfolio policies, see our Editorial Policies and the Editorial Policy Checklist.

Statistics

For all statistical analyses, confirm that the following items are present in the figure legend, table legend, main text, or Methods section.

n/a	Confirmed	
☐	☒	The exact sample size (n) for each experimental group/condition, given as a discrete number and unit of measurement
☐	☒	A statement on whether measurements were taken from distinct samples or whether the same sample was measured repeatedly
☒	☐	The statistical test(s) used AND whether they are one- or two-sided<br>*Only common tests should be described solely by name; describe more complex techniques in the Methods section.*
☒	☐	A description of all covariates tested
☒	☐	A description of any assumptions or corrections, such as tests of normality and adjustment for multiple comparisons
☐	☒	A full description of the statistical parameters including central tendency (e.g. means) or other basic estimates (e.g. regression coefficient) AND variation (e.g. standard deviation) or associated estimates of uncertainty (e.g. confidence intervals)
☒	☐	For null hypothesis testing, the test statistic (e.g. F, t, r) with confidence intervals, effect sizes, degrees of freedom and P value noted<br>*Give P values as exact values whenever suitable.*
☒	☐	For Bayesian analysis, information on the choice of priors and Markov chain Monte Carlo settings
☒	☐	For hierarchical and complex designs, identification of the appropriate level for tests and full reporting of outcomes
☒	☐	Estimates of effect sizes (e.g. Cohen's d, Pearson's r), indicating how they were calculated

Our web collection on statistics for biologists contains articles on many of the points above.

Software and code

Policy information about availability of computer code

Data collection	SUNS (ver. UTM6503), FTIR (ver. Nicolet IS50), Raman (ver. DXR2xi), XRD (ver. Siemens D5000), TGA (ver. TG209F1), SEM (ver. JSM-7600 F), TMA (ver. Q400), OpenLCA version (2.4.0, released on January 2025)
Data analysis	OriginPro 2024b (ver. 10.1.5.132), Omnic (ver. 8.0), OMNIC™xi (ver. 2.5), LAMMPS (29 Sep 2021 - Update 2)

For manuscripts utilizing custom algorithms or software that are central to the research but not yet described in published literature, software must be made available to editors and reviewers. We strongly encourage code deposition in a community repository (e.g. GitHub). See the Nature Portfolio guidelines for submitting code & software for further information.

Data

Policy information about availability of data

All manuscripts must include a data availability statement. This statement should provide the following information, where applicable:

- Accession codes, unique identifiers, or web links for publicly available datasets
- A description of any restrictions on data availability
- For clinical datasets or third party data, please ensure that the statement adheres to our policy

The data that support the findings of this study are available within this paper or included in the Supplementary Information, and from the corresponding authors upon request. Source data are provided with this paper.

Research involving human participants, their data, or biological material

Policy information about studies with human participants or human data. See also policy information about sex, gender (identity/presentation), and sexual orientation and race, ethnicity and racism.

Reporting on sex and gender	The participant's sex and gender were not considered since this study did not involve human participants.
Reporting on race, ethnicity, or other socially relevant groupings	The participant's race, ethnicity, or other socially relevant groupings were not considered since this study did not involve human participants.
Population characteristics	This study did not involve human participants, their data, or biological material. The participant's race, ethnicity, or other socially relevant groupings were not considered since this study did not involve human participants.
Recruitment	This study did not involve human participants, their data, or biological material.
Ethics oversight	This study did not involve human subjects, and thus, no ethical approval was required. The study protocol adhered to the guidelines established by the journal.

Note that full information on the approval of the study protocol must also be provided in the manuscript.

Field-specific reporting

Please select the one below that is the best fit for your research. If you are not sure, read the appropriate sections before making your selection.

- [x] Life sciences
- [ ] Behavioural & social sciences
- [ ] Ecological, evolutionary & environmental sciences

For a reference copy of the document with all sections, see nature.com/documents/nr-reporting-summary-flat.pdf

Life sciences study design

All studies must disclose on these points even when the disclosure is negative.

Sample size	No statistical methods were used to predetermine sample size. Sample size was based on preliminary experimentation. The sample size is sufficient to identify differences between groups. Details regarding sample size of all experiments are provided in the Methods section and Figure legends.
Data exclusions	No data were excluded from the analyses.
Replication	These measures were taken to verify the reproducibility, and all attempts at replication were successfully. The experiments were replicated or performed independently for 3-6 times in the manuscript and the supplementary information. The specific number of independent replicate experiments is indicated in the figure legend.
Randomization	All the experimental groups were randomly allocated.
Blinding	The investigators who assisted in data collection were blinded to group allocation during data collection and analysis.

Reporting for specific materials, systems and methods

We require information from authors about some types of materials, experimental systems and methods used in many studies. Here, indicate whether each material, system or method listed is relevant to your study. If you are not sure if a list item applies to your research, read the appropriate section before selecting a response.

Materials & experimental systems

n/a	Involved in the study	
x		Antibodies
	x	Eukaryotic cell lines
x		Palaeontology and archaeology
	x	Animals and other organisms
x		Clinical data
x		Dual use research of concern
x		Plants

Methods

n/a	Involved in the study	
x		ChIP-seq
x		Flow cytometry
x		MRI-based neuroimaging

Eukaryotic cell lines

Policy information about cell lines and Sex and Gender in Research

Cell line source(s)	L-929 cells (NCTC clone 929: CCL 1, American Type Culture Collection [ATCC])
Authentication	None of the cell lines used were authenticated.
Mycoplasma contamination	All cell lines were tested negative for mycoplasma contamination.
Commonly misidentified lines (See ICLAC register)	No misidentified cell lines were used in our study according to the results of ICLAC register.

Animals and other research organisms

Policy information about studies involving animals; ARRIVE guidelines recommended for reporting animal research, and Sex and Gender in Research

Laboratory animals	New Zealand white rabbit (Female; Healthy, young adult, nulliparous and not pregnant; Provided by Danyang Changyi experimental animal breeding Co., Ltd [Permit Code:SCXK (SU) 2021-0002]; Animals were housed in groups in cages (stainless steel cage, Suzhou Fengqiao purification equipment Co., Ltd.) identified by a card indicating the lab number, test code and first treatment date; Rabbit Diet (Beijing Keao Xieli Feed Co., Ltd.); Temperature 16-26 °C, relative humidity 40%-70%, lights 12 hours light/dark cycle; Continue use after taking a break for at least 1 week); White guinea pig (Male; Healthy, not previously used in other experimental procedures; Provided by Suzhou Hi-tech Zone Zhenhu Laboratory Animal Technology Co. , Ltd [PermitCode: SCXK (SU) 2020-0007]; Animals were housed in groups in cages (plastic cage, Suzhou Fengqiao purification equipment Co., Ltd.) identified by a card indicating the lab number and test code; Guinea Pig Diet (Beijing Keao Xieli Feed Co., Ltd.); Temperature 18-29 °C, relative humidity 40%-70%, lights 12 hours light/dark cycle; Euthanasia by CO2 inhalation).
Wild animals	This study did not involve wild animals.
Reporting on sex	This study did not involve the reporting of sex.
Field-collected samples	This study did not involve field-collected samples.
Ethics oversight	All animal procedures were in accordance with the Laboratory Animal Research Guide for the Care and Use of Laboratory Animals and approved by the Jiangsu Science Standard Medical Testing (Animal Protocol number: [IACUC22-0045], [IACUC22-0046]).

Note that full information on the approval of the study protocol must also be provided in the manuscript.

Plants

Seed stocks	This study did not involve seed stocks.
Novel plant genotypes	This study did not involve novel plant genotypes.
Authentication	This study did not involve authentication.
